# Supplementary material for: A benchmark of structural variation detection by long reads through a realistic simulated model
Source: Genome Biol. 2021 Dec 15;22:342. doi: 10.1186/s13059-021-02551-4 (PMC8672642; doi:10.1186/s13059-021-02551-4)
Supplement: Supplementary file 1 — Additional file 1 A PDF file with additional figures and tables. The file contains the following subsections: “Parameters for the structural variation callers”, “Long read simulators benchmark”, “Error profiles of long read simulators” and “Complex substitutions in NA19240”. [file 13059_2021_2551_MOESM1_ESM.pdf]

A benchmark of structural variation detection by long reads through a realistic simulated model.

Nicolas Dierckxsens[1,2]*, Tong Li [1], Joris R. Vermeesch [2] and Zhi Xie [1*]

[1] State Key Laboratory of Ophthalmology, Zhongshan Ophthalmic Center, Sun Yat-sen University, Guangzhou, 510060, China [2] Center for Human Genetics, University Hospital Leuven and KU Leuven, Leuven, Belgium

*To whom correspondence should be addressed. Email: nicolasdierckxsens@hotmail.com ; xiezhi@gmail.com

1. Parameters for the structural variation callers

1. Sniffles (v1.0.11)

We aligned the simulated reads to GRCh38 using Minimap2 (v2.17-r941). We changed the parameters of Minimap2 according to the type of simulated reads, 'minimap2 -ax map-ont' corresponds to Nanopore simulated reads and 'minimap2 -ax map-pb' corresponds to PacBio simulated reads.
The parameters of Sniffles is 'sniffles -n -1 -s 3 --genotype'.

2. SVIM (v1.3.1)

The same alignment as for Sniffles. We used SVIM to call structural variants with default parameters.

3. NanoSV (v1.2.4)

The same alignment as for Sniffles. We used NanoSV to call structural variants without 'depth_support' mode in its config file. Other parameters are default.

4. Picky (v0.2.a)

The same alignment as for Sniffles. We used the pipeline provided by the github page of Picky to call SVs (https://github.com/TheJacksonLaboratory/Picky/wiki/Using-an-Alternative-Aligner).

5. NanoVar (v1.3.8)

We changed the parameters of NanoVar according to the type of simulated reads, 'nanovar -l 50 -x ont' corresponds to Nanopore simulated reads, 'nanovar -l 50 -x pacbio-clr' corresponds to PacBio simulated reads and 'nanovar -l 50 -x pacbio-ccs' corresponds to PacBio HiFi simulated reads.

6. pbsv (v2.3.0)

We aligned simulated reads to GRCh38 using pbmm2 (v1.3.0) with default parameters. The parameters of pbsv is 'pbsv call -m 50'.

7. cuteSV (v1.0.10)

The same alignment as for Sniffles. The parameters are '--max_cluster_bias_INS 100 --diff_ratio_merging_INS 0.3 – max_cluster_bias_DEL 100 – diff_ratio_merging_DEL 0.3 – genotype -s 3

2. Long read simulators benchmark

We compared the features of 9 different long read simulators. We tested the wall time and memory consumption for the simulation of 15x coverage Nanopore or PacBio (when no Nanopore available) long reads for the human chromosome 1 (GRCh38).

Table S1 | Comparison of the features of each long read simulator and a system requirements benchmark.

	Sim-it	PBSIM	Badread	PaSS	LongISLND	DeepSimulator	Simlord	NanoSim	SURVIVOR
Error profiles	ONT, PB (RS2, Sequel2, Sequel HiFi)	PB (CCS, RS)	ONT, PB (RS2)	PB (Sequel, RS2)	/	ONT	PB (CCS, RS2)	ONT	ONT, PB (Sequel)
Train model	✔		✔	✔	✔	✔		✔	✔
Accuracy adjustment	✔	✔	✔		✔		✔		
Read length adjustment	✔	✔	✔		✔	✔	✔	✔	
Transcriptome reads								✔	
Separate haplotypes	✔								
Quality scores		✔	✔	✔	✔	✔	✔		
Sequencing depth profile	✔								✔
Wall time	35 min	5 min	938 min	12 min	31 min	1634 min	81 min	327 min	6 min
Virtual Memory	1,2 GB	0,25 GB	12,1 GB	2,5 GB	2,5 GB	14,8 GB	0,37 GB	0,43 GB	0,26 GB

3. Error profiles of long read simulators

The error profile of PBSIM (v1.0.4) is located in the installation folder and the relative path is ‘PBSIM/data/model_qc_clr’. The error profile of Badread (v0.1.5) was set ‘--error_model nanopore’ and ‘--error_model pacbio’ for the respective simulations. The error profile of PaSS is located in the installation folder (‘PaSS/E.coli/ecoli.config’). We used ‘-m pacbio_sequel -c PaSS/E.coli/ecoli.config’ for the simulation of PaSS. LongISLND (v0.9.5) does not provide an error profile so we trained error profiles using the same datasets as with Sim-it (v1.0). DeepSimulator (v1.5) provides a Nanopore error profile in the installation folder. Simlord (v1.0.3) does not provide error profiles so we changed the parameters ‘Probability for insertions, deletions, substitutions’ according to the observed values of real PacBio datasets. The command is ‘simlord -ps 0.0312 -pd 0.0309 -pi 0.0433’. We downloaded the NanoSim (v2.6.0) error profile named ‘human_NA12878_DNA_FAB49712_albacore’ from its website. We downloaded the SURVIVOR (v1.0.7) error profile named ‘NA12878_nano_error_profile_bwa.txt.zip’ and ‘HG002_Pac_error_profile_bwa.txt.zip’ from its website.

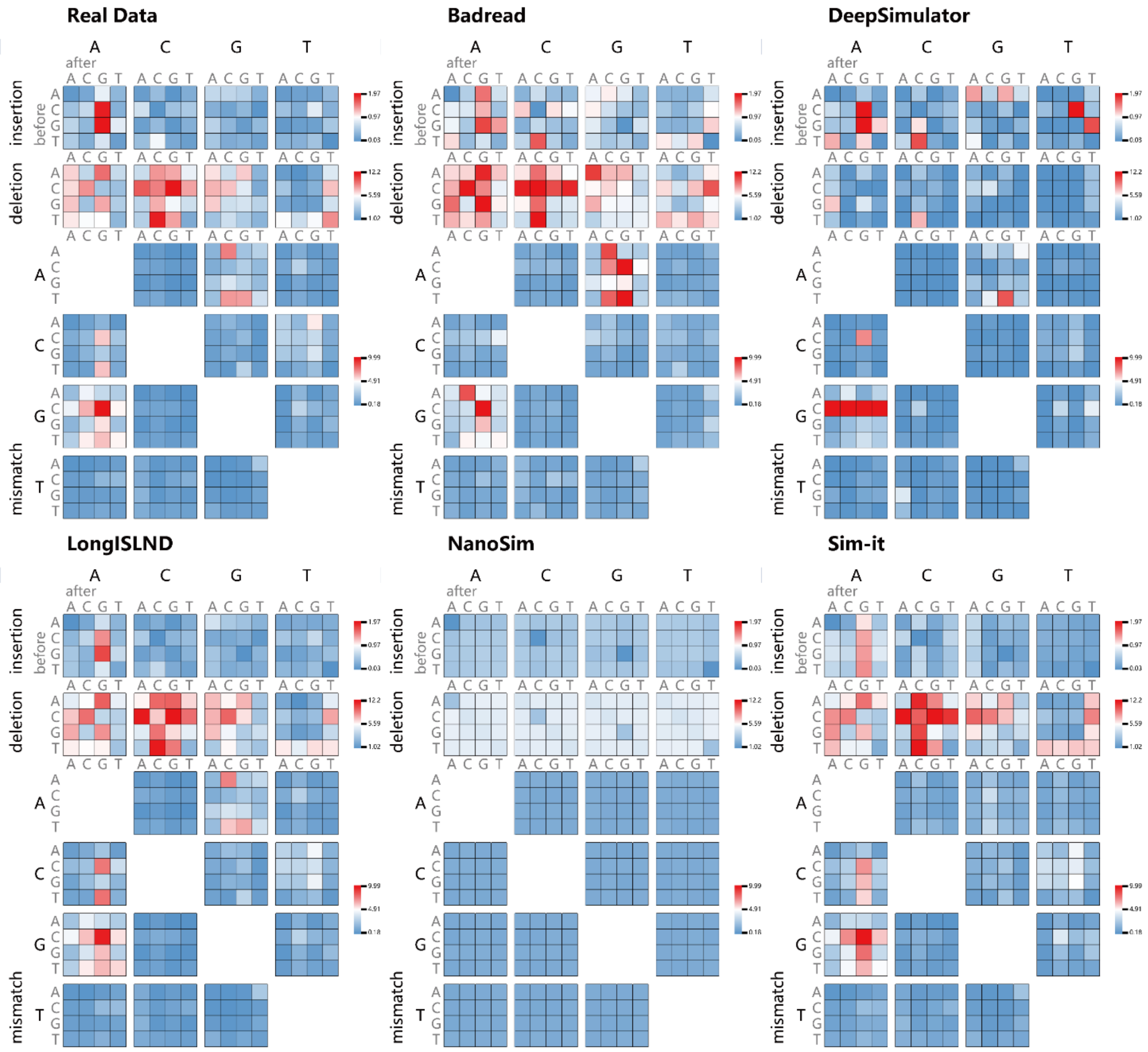

Figure S1 | Error profiles of simulated Nanopore reads from 5 different long read simulators.

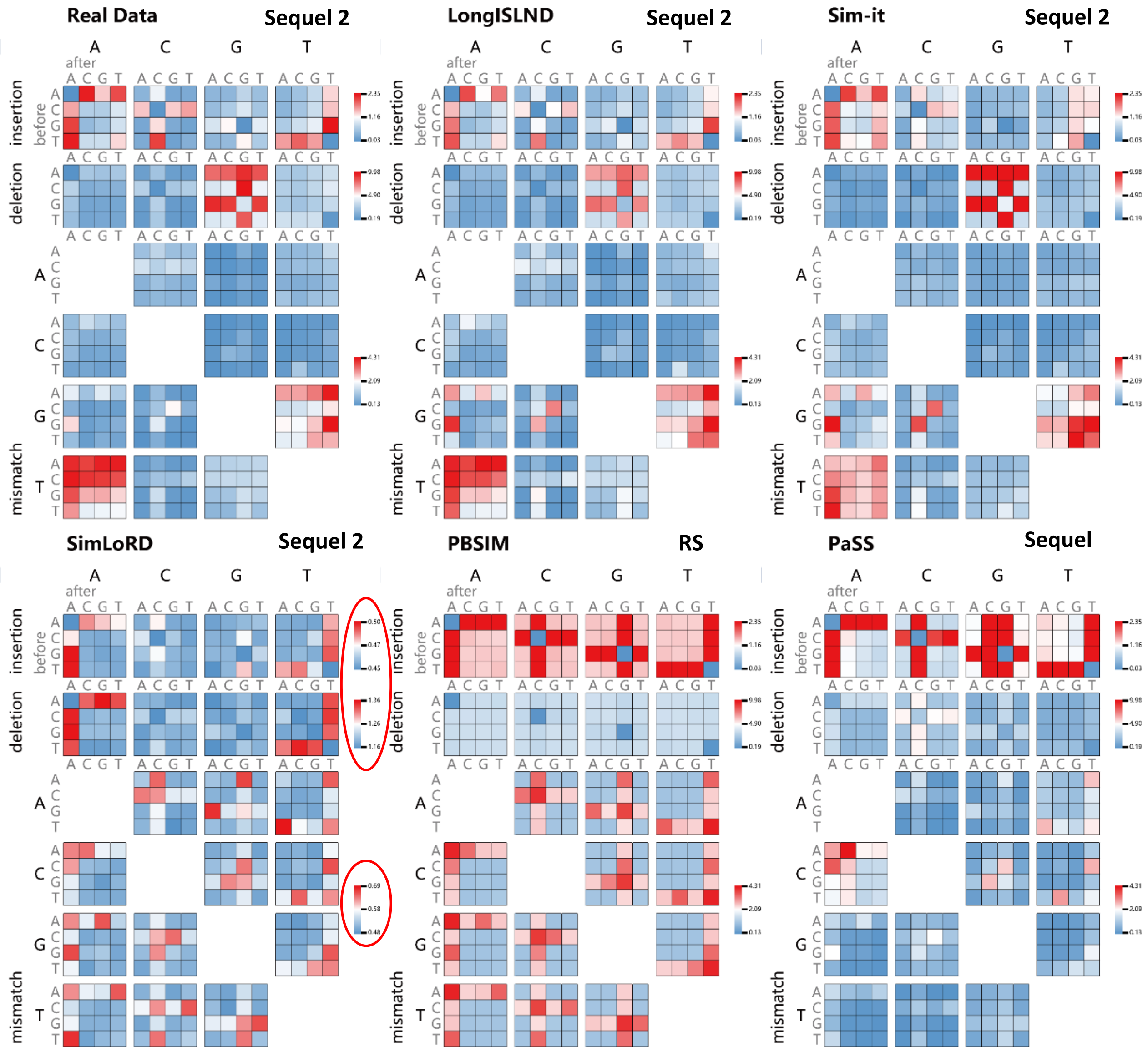

Figure S2 | Error profiles of simulated PacBio reads from 5 different long read simulators. The error rate for Simlord is much lower compared to the real data, we therefore adjusted the ratios to visualize the error profile.

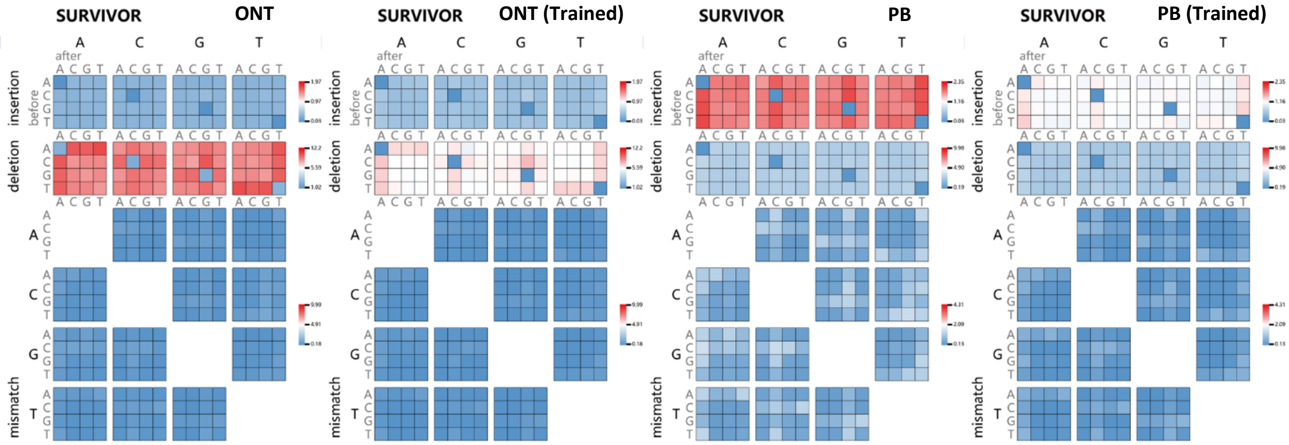

Figure S3 | Error profiles of simulated ONT and PacBio reads from the SURVIVOR simulator. For both ONT and PacBio, we used both the provided error profile and a trained error profile from the data sets we used to train Sim-it.

Table S2 | Statistics of the simulated reads for Nanopore for chromosome 1 of GRCh38.

	Match rate (%)	Insertion rate (%)	Deletion rate (%)	Substitution rate (%)	Totel error rate (%)	Average length (bp)
Real data	88,21	1,95	5,62	4,22	11,79	18.269
Sim-it	87,63	2,07	5,83	4,47	12,37	17.785
Badread	86,84	2,12	6,70	4,34	13,16	14.222
LongISLND	88,15	1,81	5,73	4,31	11,85	9.129
DeepSimulator	93,43	2,20	1,79	2,58	6,57	7.986
SURVIVOR	88,51	1,32	8,94	1,23	11,49	5.639
NanoSim	88,70	2,60	4,78	3,92	11,30	13.102

Table S3 | Statistics of the simulated reads of PacBio Sequel II for E. coli K12 substrain MG1655.

	Match rate (%)	Insertion rate (%)	Deletion rate (%)	Substitution rate (%)	Totel error rate (%)	Average length (bp)
Real data	89,50	4,29	3,09	3,12	10,50	11.478
Sim-it	89,06	4,29	3,00	3,65	10,94	11.839
PBSIM	82,02	9,37	3,02	5,59	17,98	3.035
Badread	87,25	3,67	5,34	3,74	12,75	14.939
PaSS	86,30	8,69	2,27	2,74	13,70	9.294
LongISLND	89,91	3,88	2,64	3,57	10,09	10.100
Simlord	95,52	1,76	1,11	1,61	4,48	8.155
SURVIVOR	89,31	6,57	2,48	1,64	10,69	6.435

Table S4 | Comparison of insertion and deletion lengths, and context-specific error patterns for mismatches and indels between simulated reads and the real sequencing data. Euclidean distances were calculated to compare simulated data for different simulators and real sequencing data. For each column and each dataset a color heatmap was adopted with blue indicating the most accurate caller.

	Methods	Mismatch patterns	Deletion patterns	Insertion patterns	Deletion lengths	Insertion lengths
		Euclidean distance	Euclidean distance	Euclidean distance	Euclidean distance	Euclidean distance
Nanopore (9.4.1) chromosome 1 of GRCh38	Sim-it	11.82	9.02	1.8	137.54	37.04
	Badread	15.11	13.35	1.92	497.99	337.3
	LongISLND	2.92	5.97	0.72	939.08	44.26
	DeepSimulator	24.5	34.31	6.48	973.54	107.58
	SURVIVOR	28.73	35.51	2.67	2574.67	143.59
	NanoSim	23.8	23.5	2.68	326.32	137.54
PacBio Sequel II E. coli K12 substrain MG1655	Sim-it	8.2	12.43	3.25	303.76	36.87
	PBSIM	20.04	20.5	12	101.3	2398.47
	Badread	16.69	32.78	3.15	852.44	299.44
	PaSS	16.39	22.1	9.77	551.74	1560.25
	LongISLND	4.04	5.39	1.06	180.57	147.26
	Simlord	15.33	25.64	5.82	1005.05	780.34
	SURVIVOR	14.88	20.37	7.73	279.73	1939.52

Table S5 | System requirements for each of the 6 tested SV callers. Each tool was used on the simulated dataset of Nanopore with 20x coverage and the GIAB dataset. SVIM cannot be run with multiple threads. Other SV callers are run with 24 threads

		Sniffles	cuteSV	pbsv	NanoVar	NanoSV	SVIM	combiSV	SURVIVOR
ONT simulted (20x)	Runtime (min)	64.0	5.0	172.0	62.0	66.0	31.0	<1	<1
	Peak memory (GB)	3.3	15.6	13,3	4.0	8.5	1.0	<1	<1
ONT GIAB (45x)	Runtime (min)	288.0	54.0	201.0	169.0	2913.0	199.0	2.0	<1
	Peak memory (GB)	10.9	23.1	65.4	53.2	39.9	2.8	<1	<1

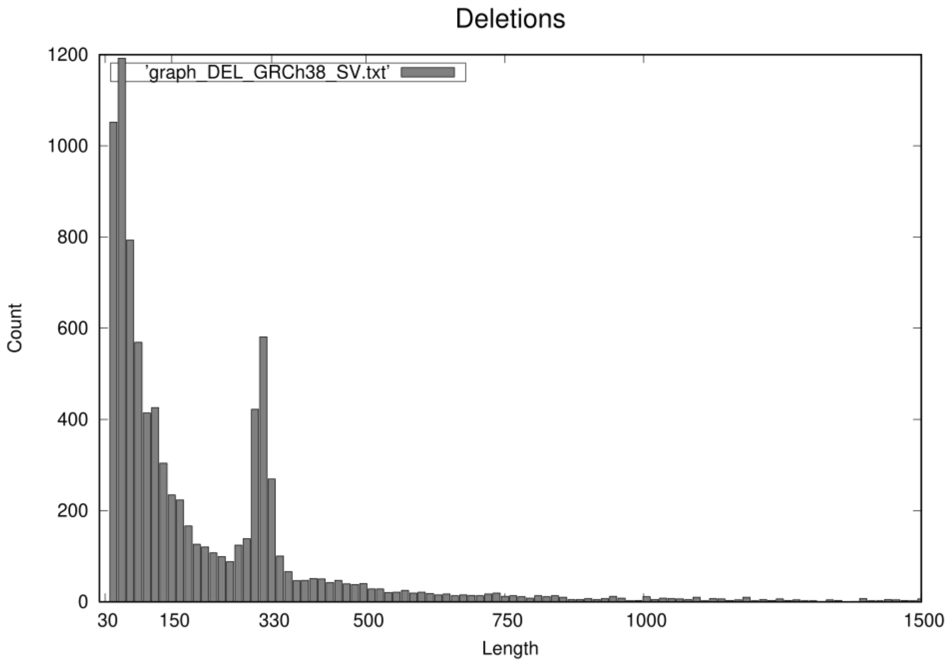

Figure S4 | Graphical output of the deletion length distribution from the structural variation simulation of the 24,600 SVs derived from sample NA19240 of dbVAR nstd152.

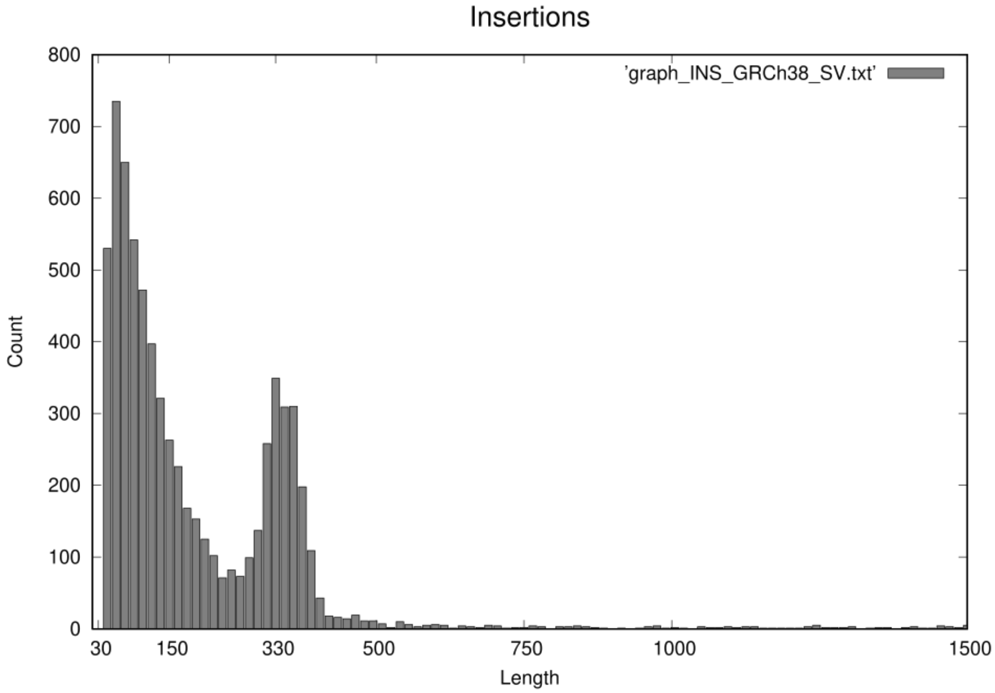

Figure S5 | Graphical output of the insertion length distribution from the structural variation simulation of the 24,600 SVs derived from sample NA19240 of dbVAR nstd152.

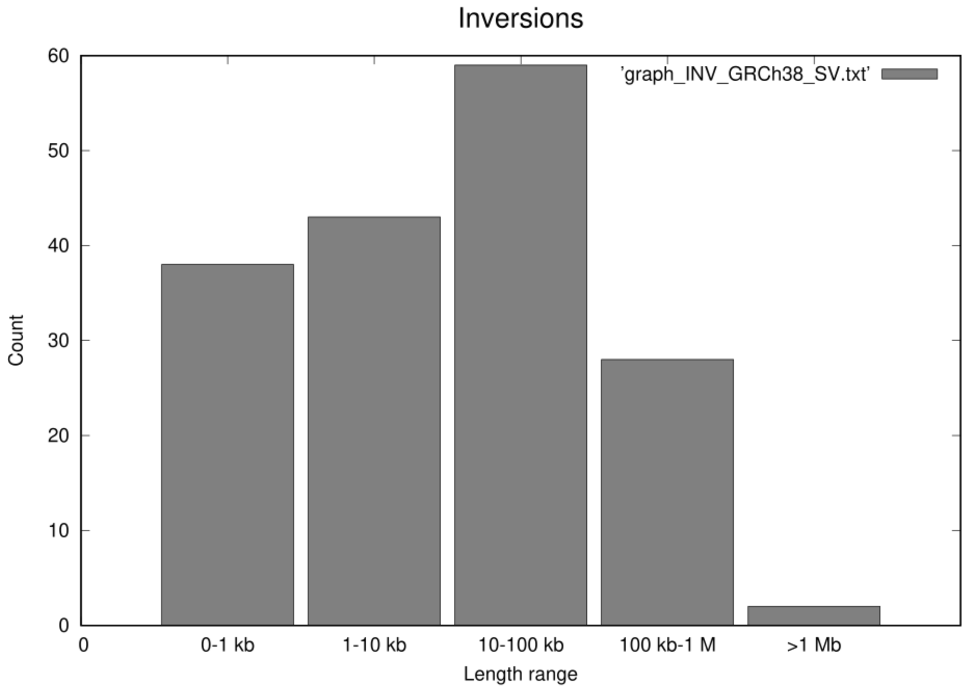

Figure S6 | Graphical output of the inversion length distribution from the structural variation simulation of the 24,600 SVs derived from sample NA19240 of dbVAR nstd152.

Table S6 | Comparison between combiSV and SURVIVOR for 9 combinations of three SV callers on a simulated Nanopore dataset of 20x and the GIAB reference dataset (Nanopore). The highest scores between combiSV and SURVIVOR are indicated in grey.

			cuteSV Sniffles NanoSV	cuteSV Sniffles NanoVar	cuteSV Sniffles SVIM	cuteSV pbsv NanoSV	cuteSV pbsv NanoVar	cuteSV pbsv SVIM	cuteSV pbsv Sniffles	cuteSV NanoSV SVIM	SVIM NanoSV NanoVar
Simulation (Nanopore)	combiSV	Recall	81.3%	80.7%	80.6%	79.7%	79.4%	79.8%	80.4%	79.3%	80.1%
		Precision	98.0%	98.6%	98.4%	98.7%	98.8%	98.7%	98.5%	98.7%	97.5%
		**F-score**	**88.9%**	**88.8%**	**88.6%**	**88.2%**	**88.0%**	**88.2%**	**88.6%**	**87.9%**	**87.9%**
		Perfect matches	8.0%	7.9%	8.0%	31.6%	31.6%	31.6%	31.7%	8.0%	3.6%
		Position score	85.2%	85.2%	85.2%	88.0%	88.0%	88.1%	88.6%	84.7%	85.4%
		Length score	87.9%	87.9%	87.9%	91.7%	91.8%	91.8%	92.0%	87.9%	87.4%
		Type score	92.7%	93.8%	93.8%	94.2%	94.3%	94.3%	93.7%	94.4%	94.2%
		Genotype score	95.2%	94.0%	94.7%	94.9%	95.2%	95.2%	95.3%	95.8%	95.1%
		**Total score**	**72.6%**	**72.0%**	**72.0%**	**71.8%**	**71.7%**	**71.9%**	**72.5%**	**69.9%**	**69.6%**
	SURVIVOR	Recall	79.3%	75.2%	77.8%	77.9%	75.7%	77.9%	72.0%	78.5%	78.7%
		Precision	97.9%	98.4%	97.7%	98.4%	98.8%	97.8%	98.4%	97.5%	97.7%
		**F-score**	**87.6%**	**85.2%**	**86.6%**	**87.0%**	**85.7%**	**86.7%**	**83.2%**	**87.0%**	**87.1%**
		Perfect matches	6.0%	4.9%	5.3%	6.0%	5.0%	5.4%	18.6%	0.4%	2.8%
		Position score	81.2%	78.5%	78.6%	80.4%	78.3%	78.4%	88.0%	85.0%	85.0%
		Length score	86.7%	88.6%	86.8%	87.5%	89.7%	88.2%	88.4%	86.2%	87.4%
		Type score	79.9%	94.6%	94.2%	81.0%	94.3%	94.2%	93.6%	49.8%	50.4%
		Genotype score	80.7%	92.6%	93.2%	82.3%	94.6%	94.5%	92.5%	51.2%	51.8%
		**Total score**	**63.3%**	**63.8%**	**65.3%**	**62.9%**	**65.0%**	**65.8%**	**63.7%**	**54.1%**	**54.8%**
GIAB (Nanopore)	combiSV	Recall	93.5%	93.6%	93.6%	94.4%	95.1%	95.4%	95.4%	93.8%	85.0%
		Precision	93.9%	93.6%	93.3%	92.8%	92.1%	91.6%	92.7%	90.1%	91.7%
		**F-score**	**93.7%**	**93.6%**	**93.5%**	**93.6%**	**93.6%**	**93.4%**	**94.0%**	**91.9%**	**88.2%**
		Perfect matches	0.4%	0.4%	0.4%	25.6%	25.4%	25.4%	25.4%	6.6%	5.2%
		Position score	66.3%	66.2%	66.2%	72.7%	72.3%	72.3%	72.3%	66.3%	70.1%
		Length score	79.7%	79.7%	79.7%	86.6%	86.2%	86.0%	85.5%	79.3%	80.7%
		Type score	98.8%	98.7%	98.8%	99.2%	98.7%	98.7%	98.7%	98.7%	95.6%
		Genotype score	97.4%	91.7%	97.1%	97.2%	96.0%	96.4%	96.9%	94.1%	92.5%
		**Total score**	**70.3%**	**69.0%**	**69.7%**	**73.6%**	**72.8%**	**72.4%**	**73.7%**	**65.6%**	**61.8%**
	SURVIVOR	Recall	80.9%	91.6%	93.8%	81.3%	91.0%	94.2%	93.5%	93.8%	90.8%
		Precision	94.1%	92.6%	77.4%	95.3%	92.9%	77.8%	93.2%	78.1%	90.4%
		**F-score**	**87.0%**	**92.1%**	**84.8%**	**87.8%**	**92.0%**	**85.2%**	**93.3%**	**85.2%**	**90.6%**
		Perfect matches	2.9%	1.7%	1.8%	2.6%	1.6%	1.8%	2.3%	1.7%	1.0%
		Position score	64.6%	60.0%	57.2%	64.6%	60.7%	57.4%	60.1%	58.6%	60.8%
		Length score	79.5%	81.5%	78.5%	81.9%	83.1%	80.8%	82.7%	78.3%	79.5%
		Type score	97.9%	97.9%	98.6%	98.6%	94.6%	98.8%	98.7%	98.5%	98.0%
		Genotype score	86.6%	77.1%	79.8%	90.8%	90.1%	91.0%	88.4%	89.1%	84.7%
		**Total score**	**58.6%**	**61.7%**	**42.3%**	**61.1%**	**63.9%**	**45.7%**	**66.1%**	**45.5%**	**60.0%**

4. Complex substitutions in NA19240

The recall of complex substitutions (CSUB) are significantly higher for the real PacBio dataset (60%) of NA19240 than for our simulated datasets (1-20%). Because we expected a drop in recall we examined the alignment of 27 CSUBs manually with IGV. We selected only homozygous CSUBs to simplify the interpretation of the alignments. CSUBs were not selected on any other criteria, we selected 12 random homozygous CSUBs from chromosome 1 and 15 random homozygous CSUBs of chromosome 2. For the simulated CSUBs, the length of the deleted sequences were always the same as the length of the inserted sequences. This is not necessarily the case for the real CSUBs, which could partially explain the discrepancy between the recall values and different alignment patterns. Nevertheless, several of the CSUBs we examined were in fact deletions or insertions that were incorrectly categorized as a CSUB. From only examining the alignments, we could only confirm one CSUB out of the 27 potential CSUBs as a true CSUB. For 6 presumed CSUBs, we aligned and inspected several individual PacBio reads separately. When the alignment does not show any SV at the given position, it also possible that called position was inaccurate. For each of the screenshots of IGV, the top alignment is the simulation and the bottom the true dataset of NA19240.

Figure 7: We did not observe a SV at this position by inspecting the alignments.

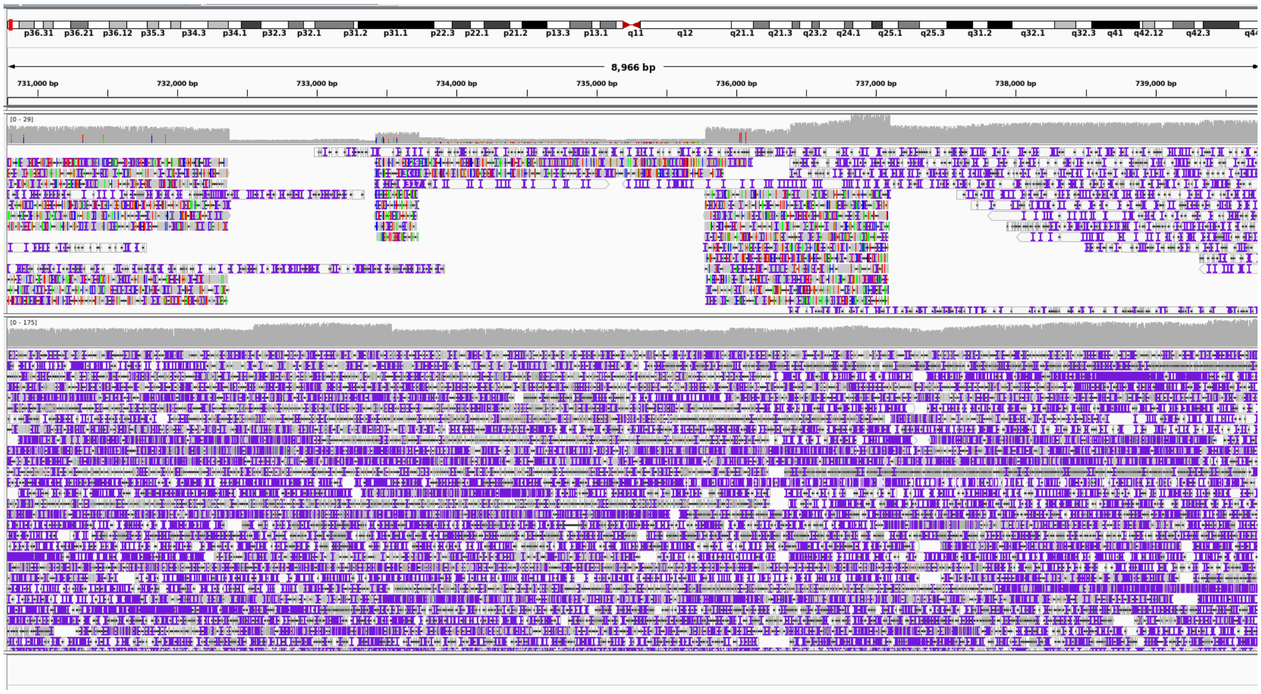

Figure S7 | CSUB chromosome 1, position 732377, length 3402 bp

Figure 8: This is the only alignment that visually resembles a theoretical CSUB. SV callset ‘nstd152’ called 3 CSUBs of 61 bp within a region of 400 bp. This region is a tandem repeat region and when we individually aligned several PacBio reads, we found that there are two different haplotypes. Both haplotypes have a shorter tandem repeat region compared to the reference and for one haplotype we also observed an insertion of around 100 bp and can there be categorized as a heterozygous CSUB.

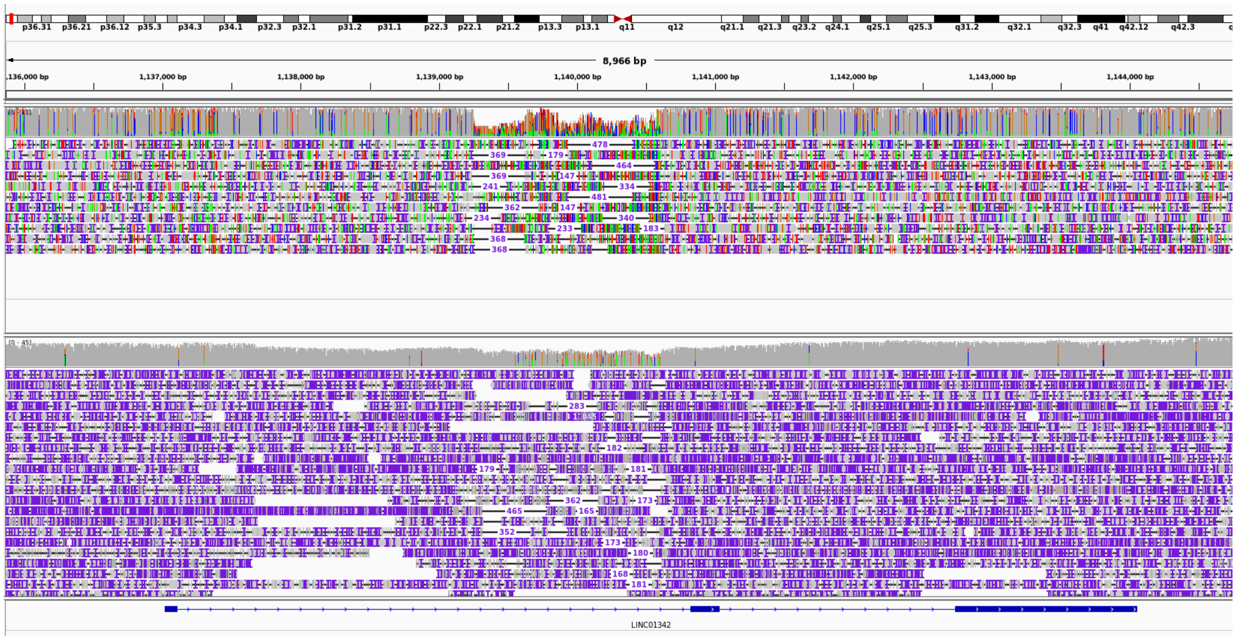

Figure S8 | CSUB chromosome 1, position 1140181, length 61 bp

Figure 9 & 10: This SV is a confirmed deletion, as can be seen in the overall alignment and the individual PacBio alignments.

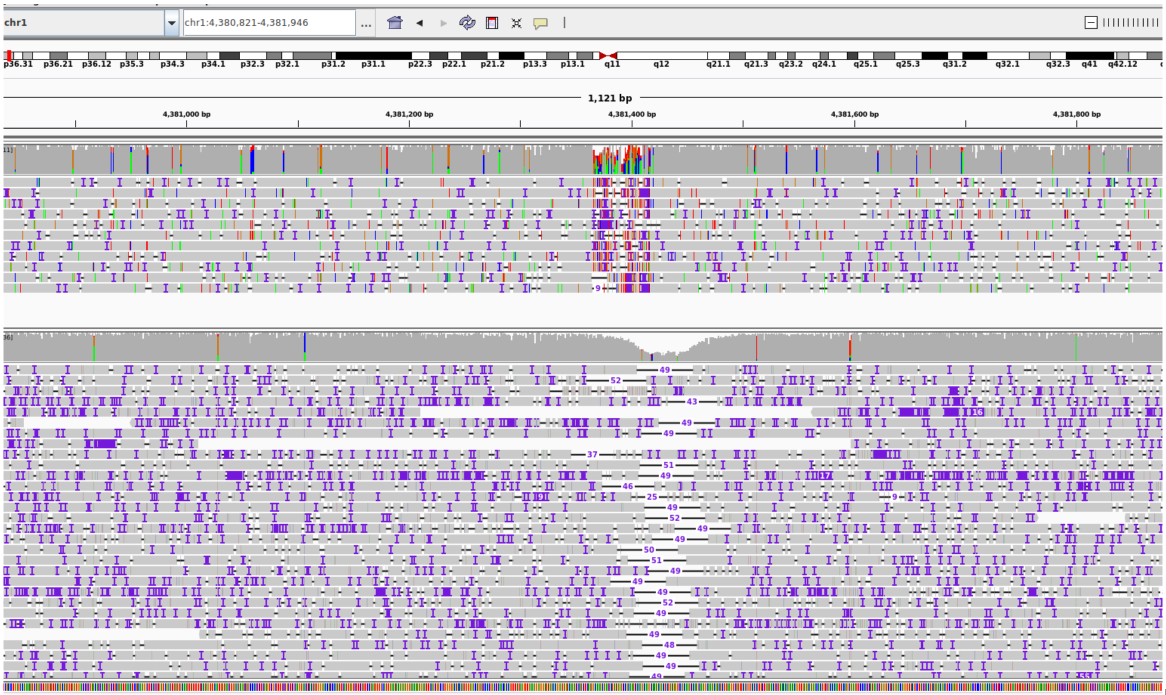

Figure S9 | CSUB chromosome 1, position 4381366, length 51 bp.

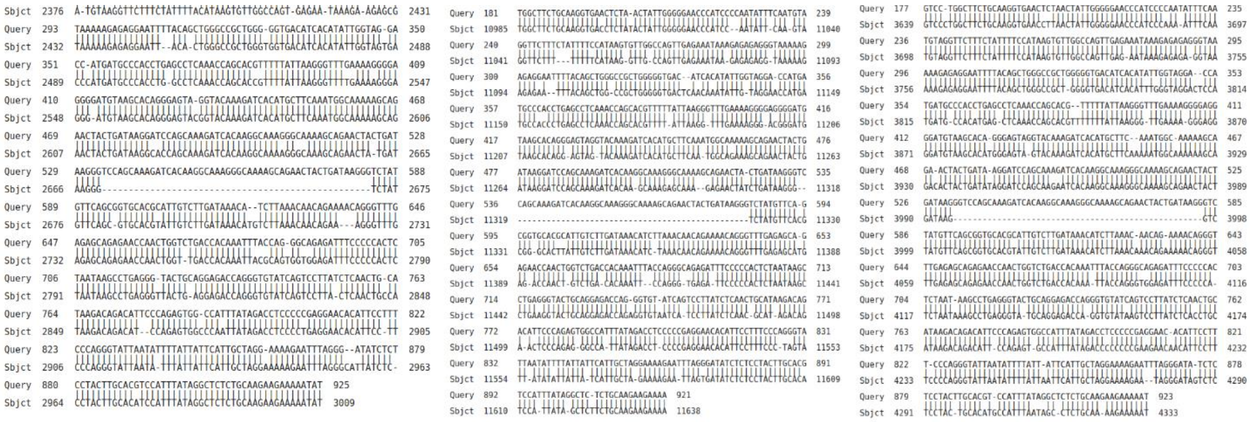

Figure S10 | Individual alignment of three PacBio reads for ‘CSUB chromosome 1, position 4381366, length 51 bp’.

Figure 11: Several reads show insertions around this position.

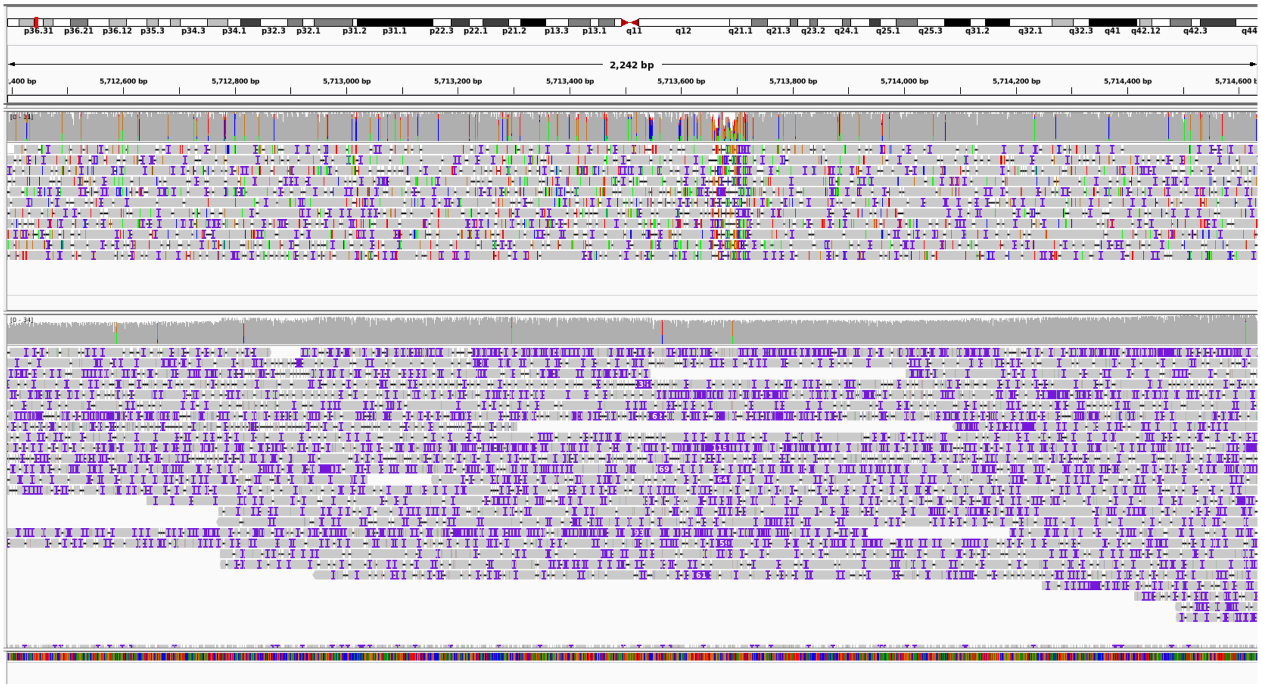

Figure S11 | CSUB chromosome 1, position 5713658, length 62 bp

Figure 12 & 13: We did not observe a SV at either position by inspecting the alignments.

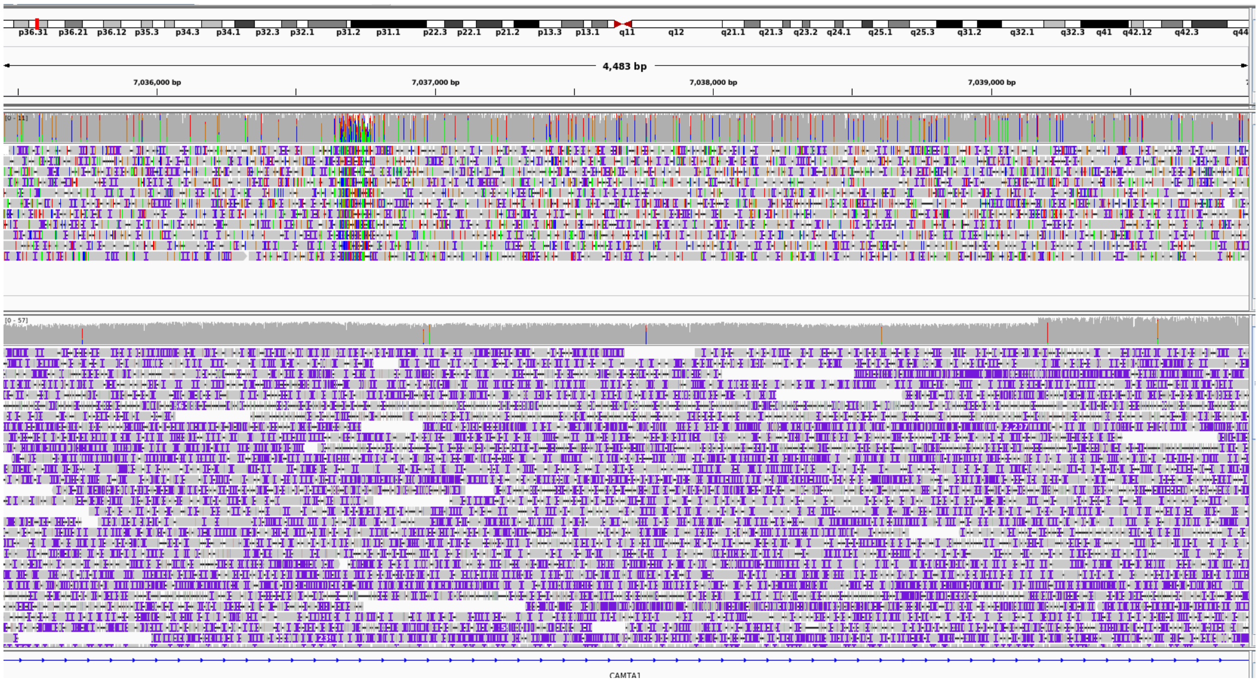

Figure S12 | CSUB chromosome 1, position 7036659, length 119 bp

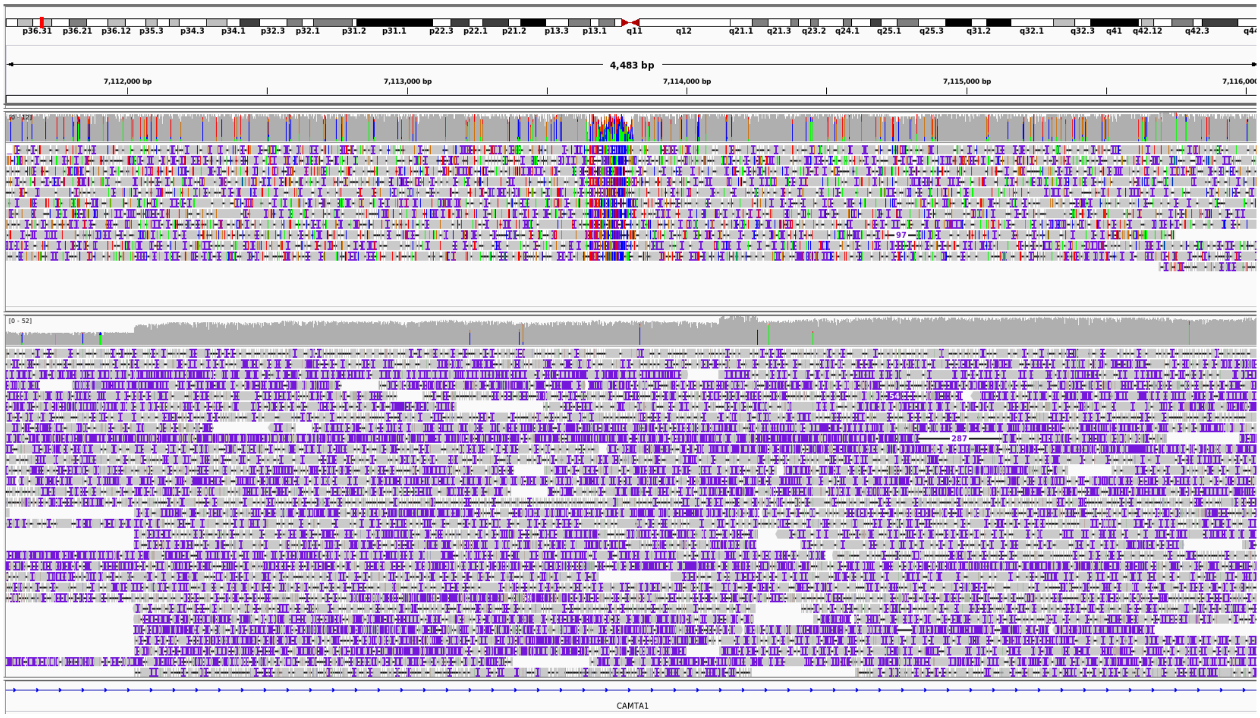

Figure S13 | CSUB chromosome 1, position 7113644, length 165 bp

Figure 14 & 15: This SV is a confirmed insertion, as can be seen in the overall alignment and the individual PacBio alignments.

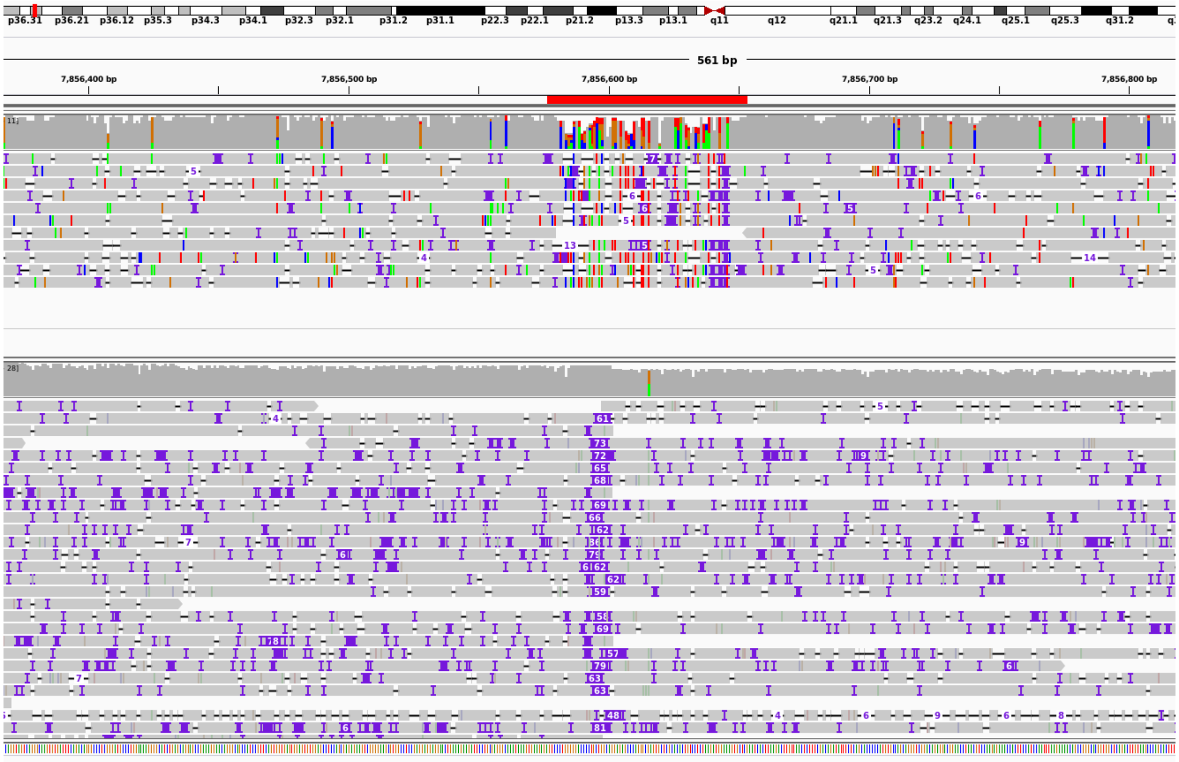

Figure S14 | CSUB chromosome 1, position 7856583, length 62 bp

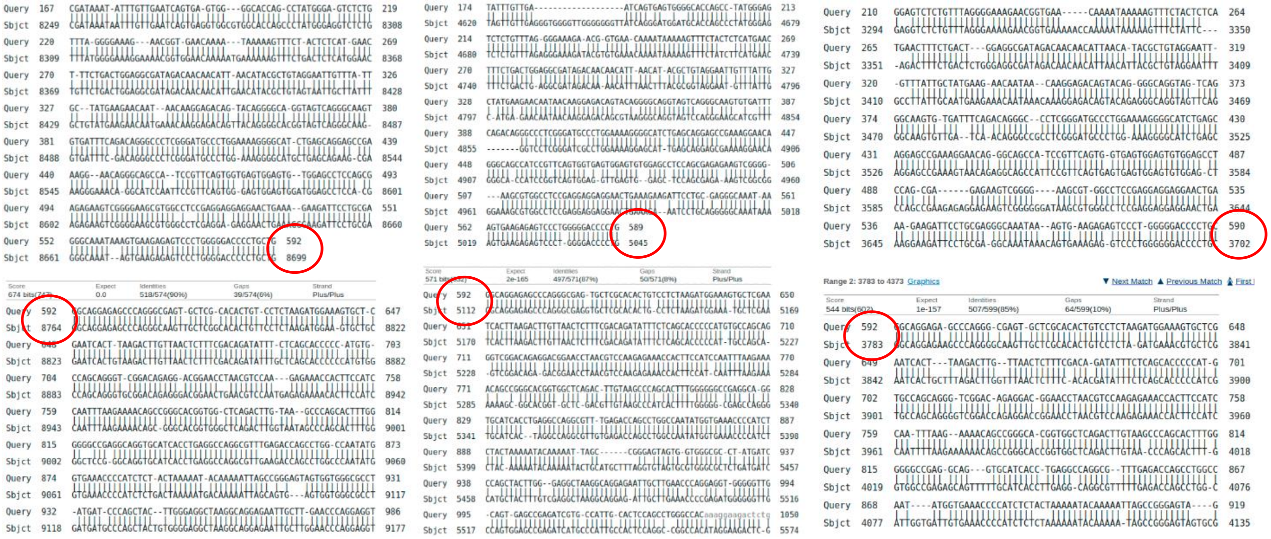

Figure S15 | Individual alignment of three PacBio reads for ‘CSUB chromosome 1, position 7856583, length 62 bp’.

Figure 16 & 17: This SV is a confirmed deletion, as can be seen in the overall alignment and the individual PacBio alignments. By comparing individual PacBio reads with the reference we concluded that the first duplicated sequence was deleted in the NA19240 (first yellow sequence in Figure 13).

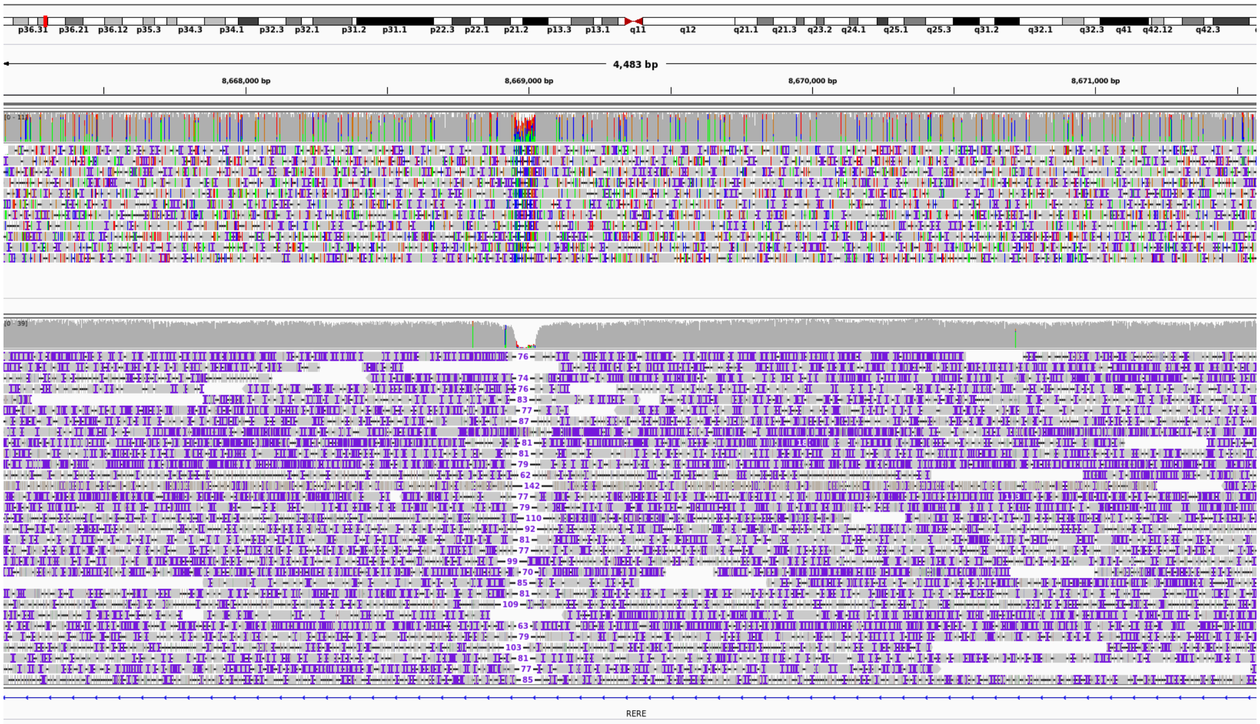

Figure S16 | CSUB chromosome 1, position 8668948, length 76 bp

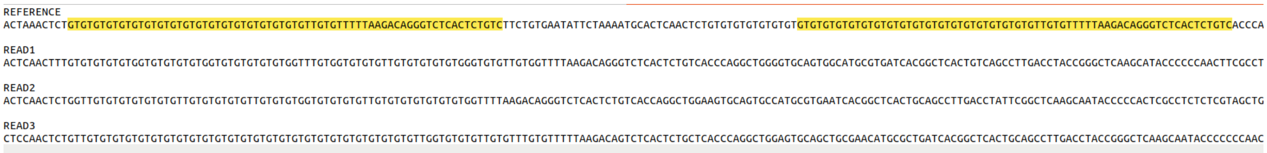

Figure S17 | Individual alignment of three PacBio reads for 'CSUB chromosome 1, position 8668948, length 76 bp'.

Figure 18 & 19: This SV is a confirmed insertion, as can be seen in the overall alignment and the individual PacBio alignments. Although it seems this insertion was heterozygous, as half of the reads do not show any inserted sequence.

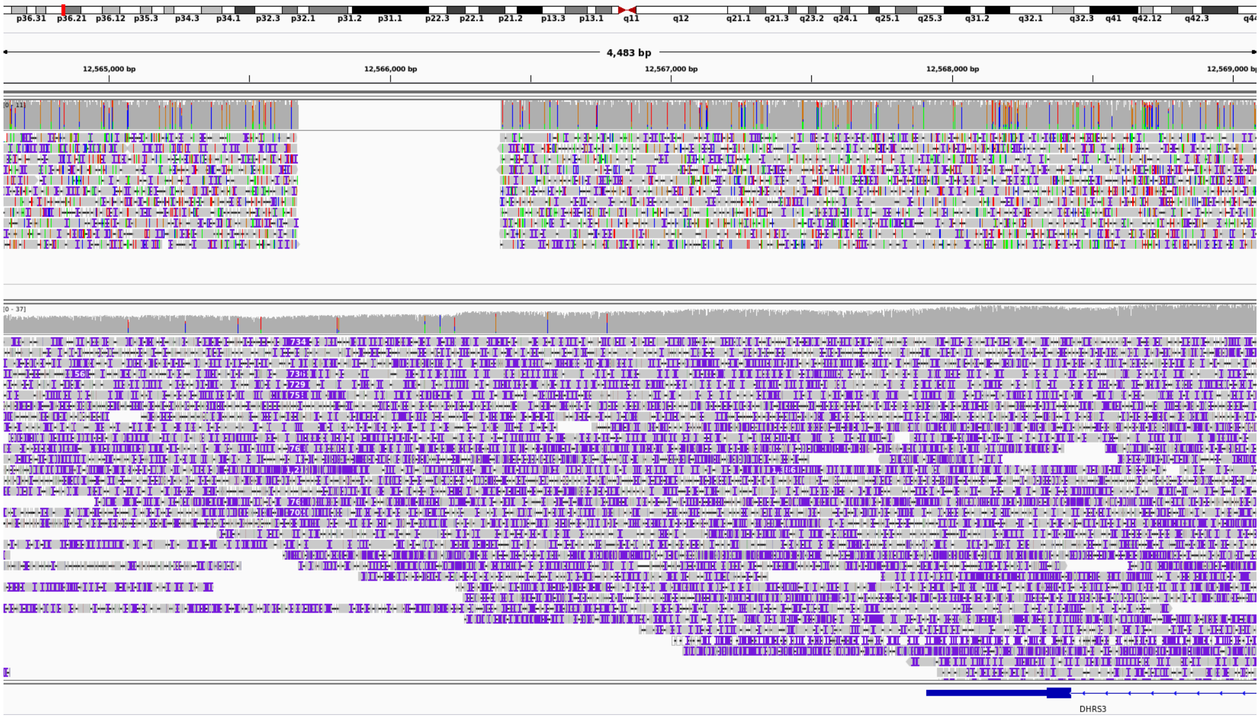

Figure S18 | CSUB chromosome 1, position 12565675, length 719 bp

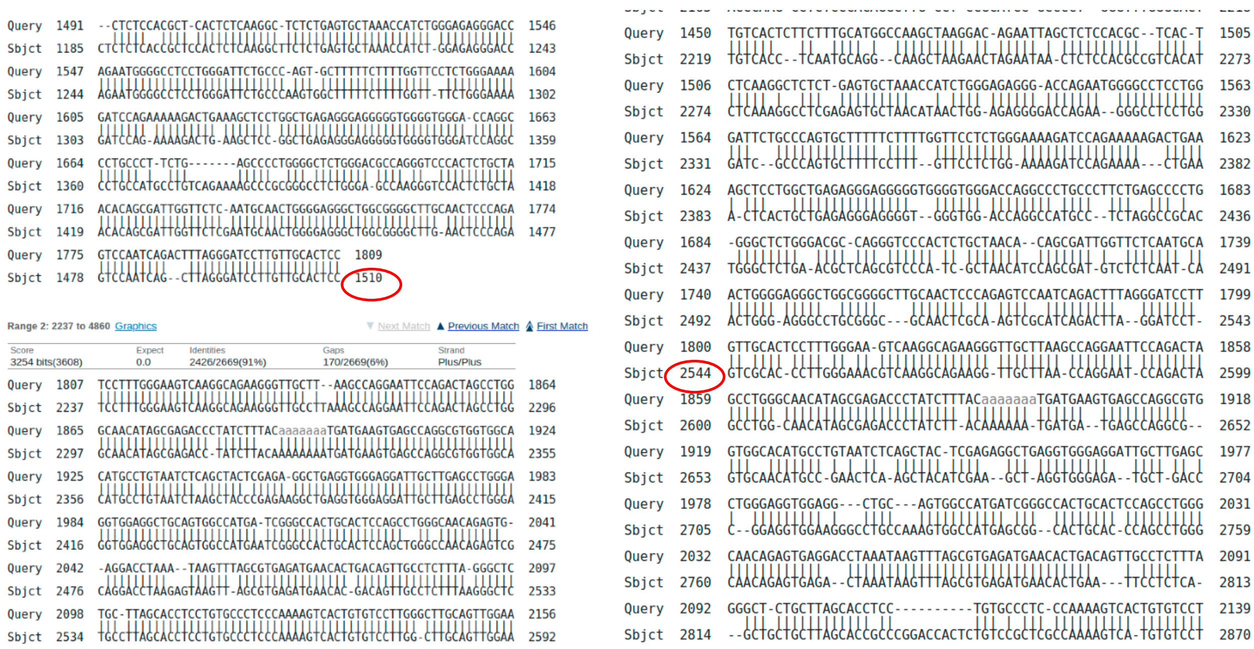

Figure S19 | Individual alignment of two PacBio reads for ‘CSUB chromosome 1, position 12565675, length 719 bp’.

Figure 20: Unconfirmed SV.

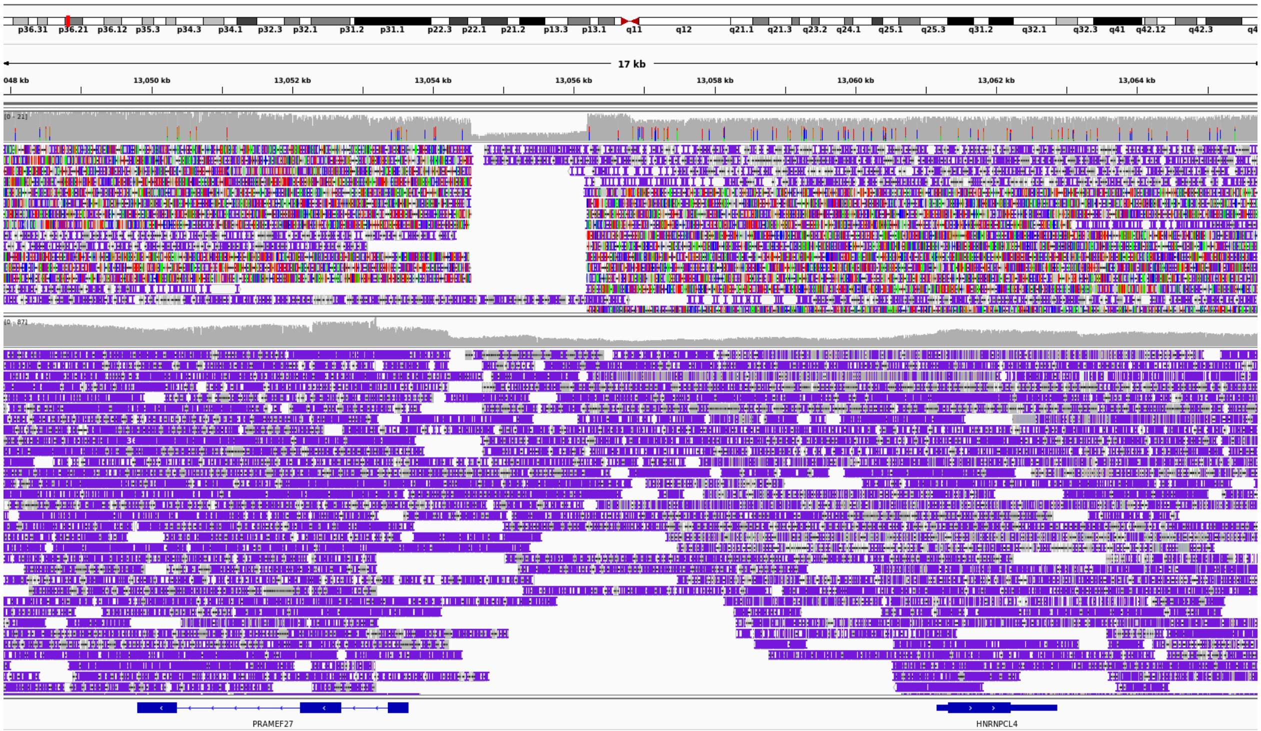

Figure S20 | CSUB chromosome 1, position 13054543, length 1646 bp

Figure 21: We observed an insertion, although this is a duplicated region which makes alignments less reliable.

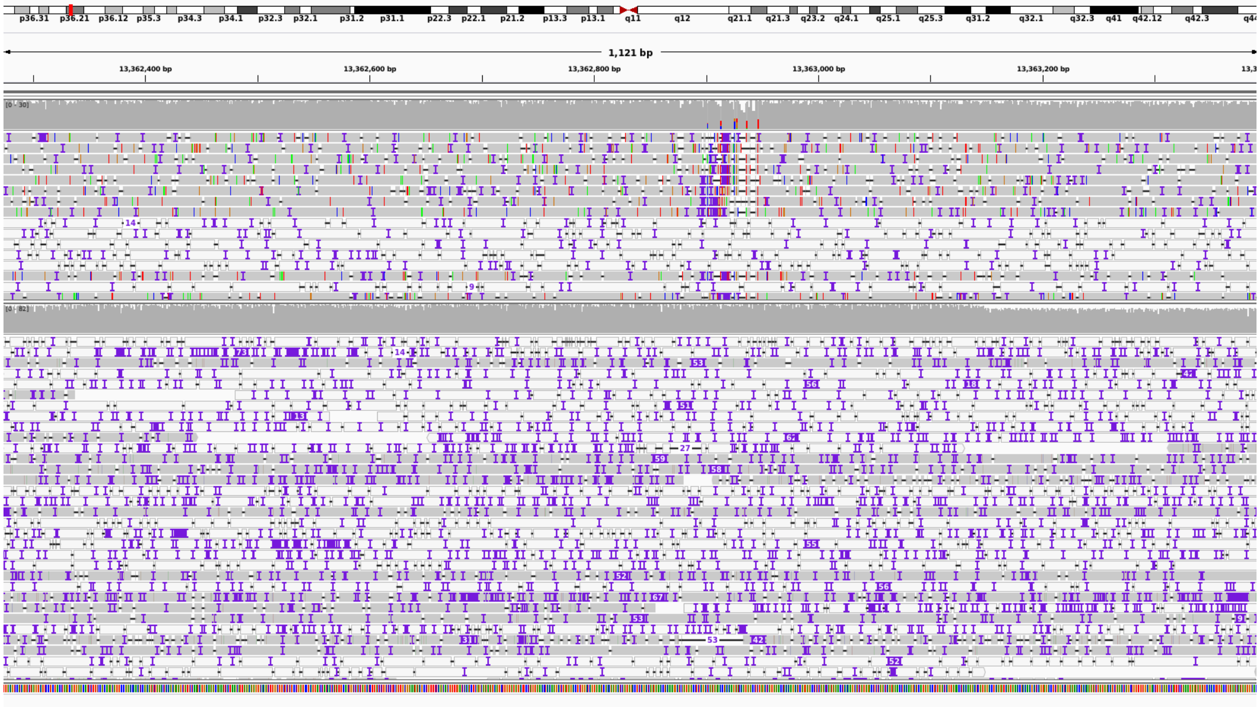

Figure S21 | CSUB chromosome 1, position 13362897, length 53 bp

Figure 22: We observed an heterozygous insertion.

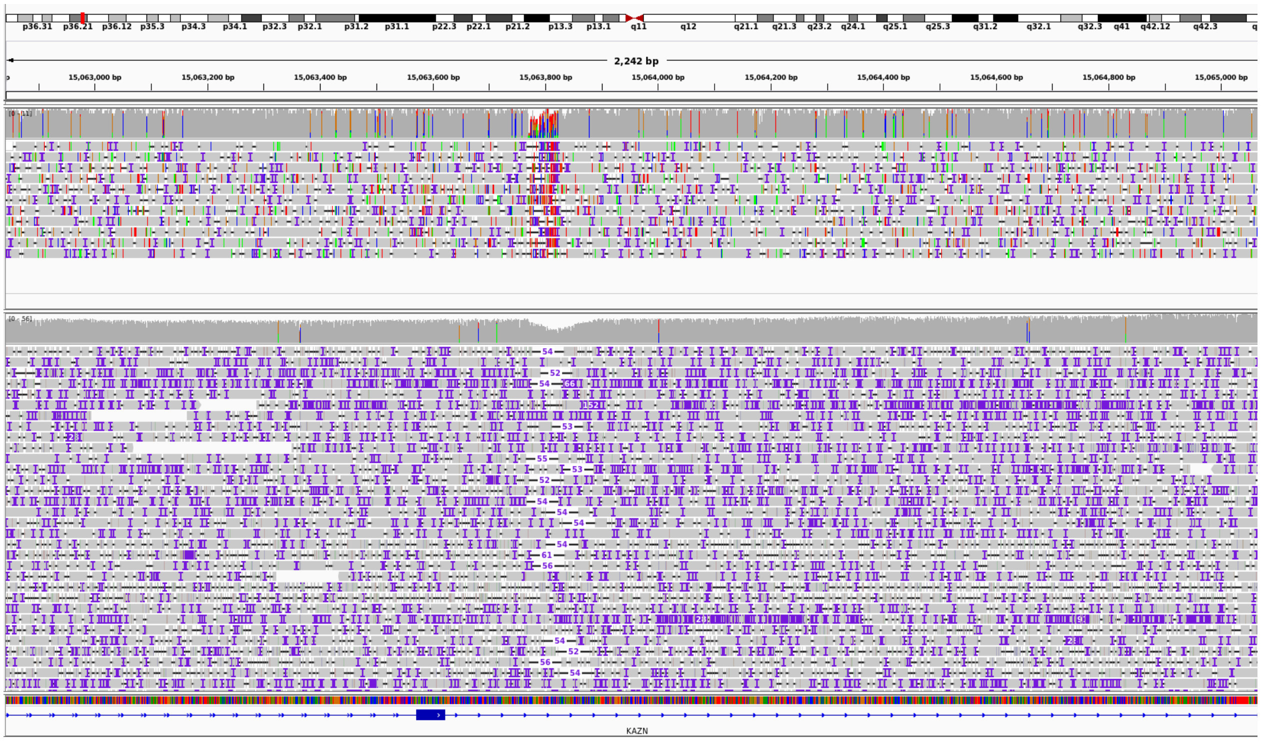

Figure S22 | CSUB chromosome 1, position 6935636, length 64 bp

Figure 23: We observed a deletion.

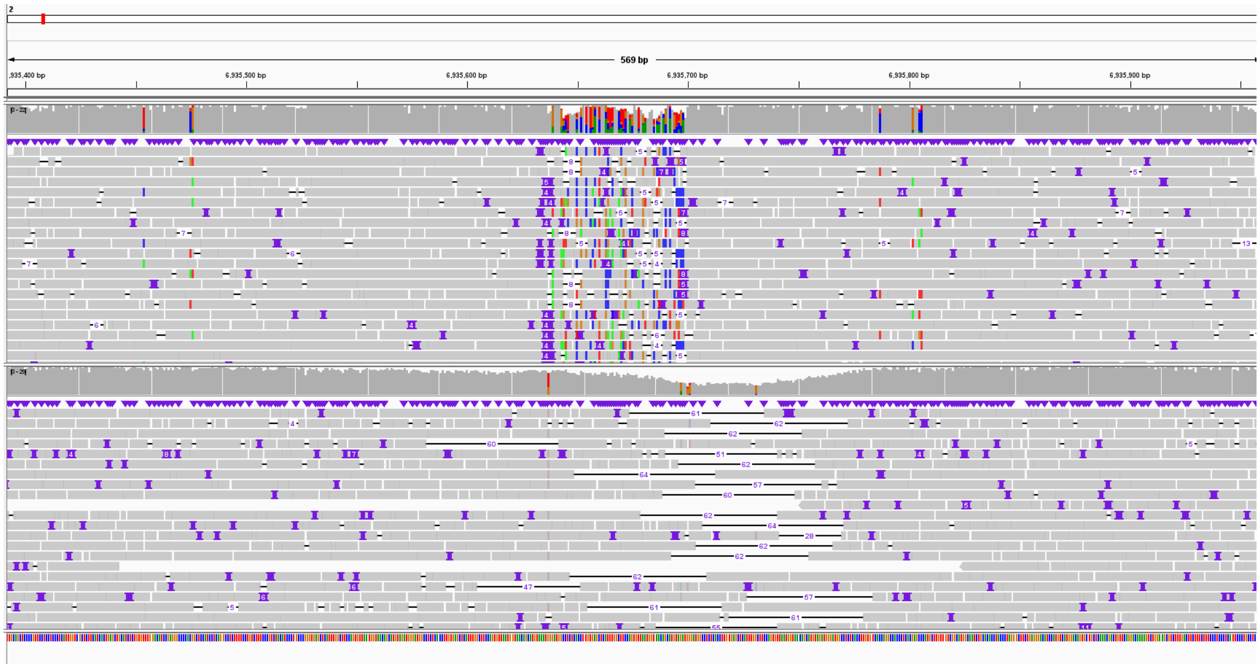

Figure S23 | CSUB chromosome 2, position 6935636, length 64 bp

Figure 24 - 26: We did not observe a SV at any of the positions by inspecting the alignments.

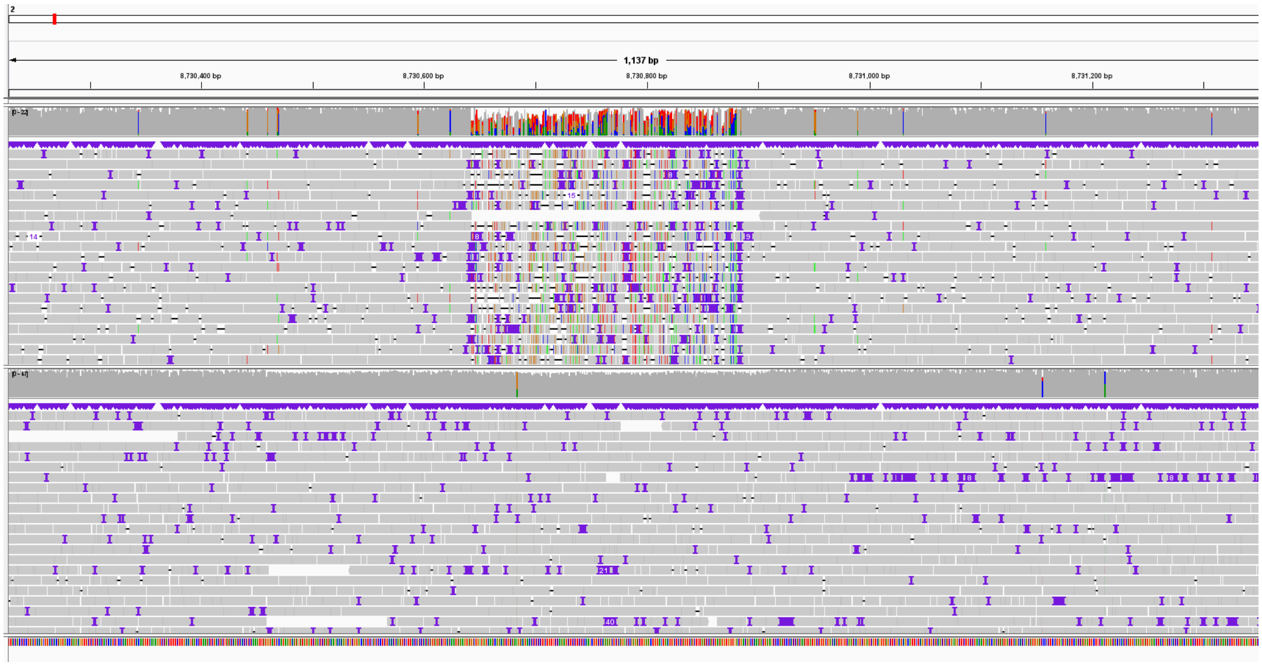

Figure S24 | CSUB chromosome 2, position 8730642, length 242 bp

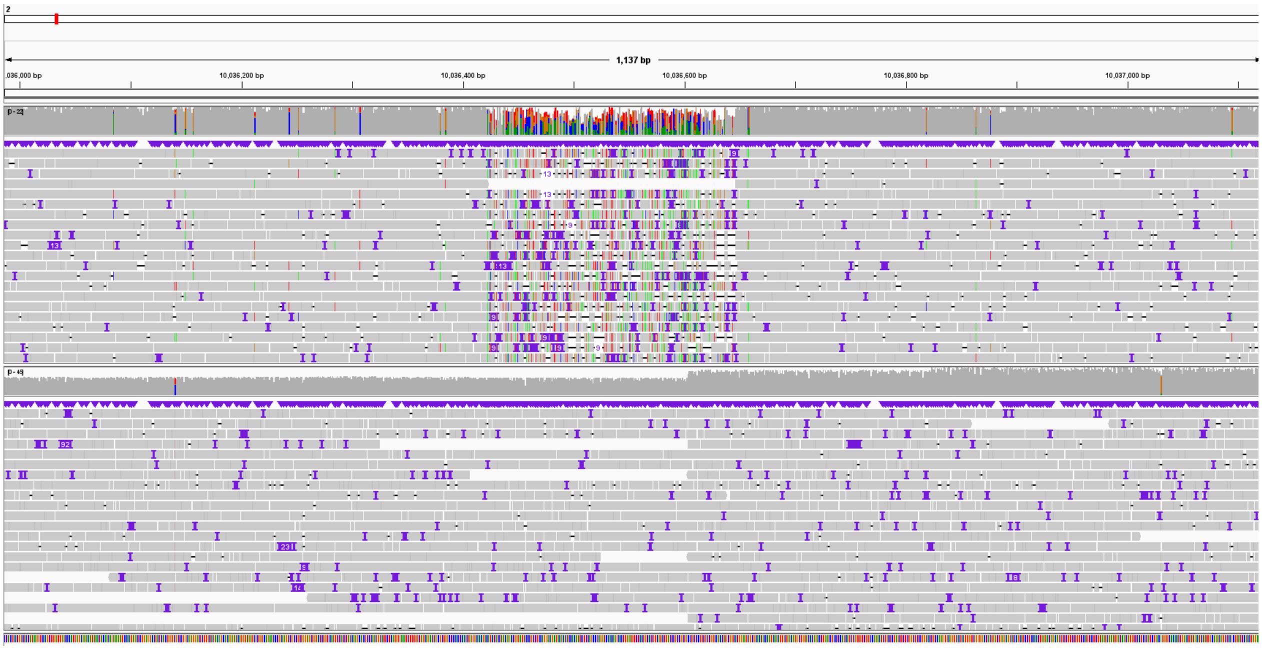

Figure S25 | CSUB chromosome 2, position 10036422, length 225 bp

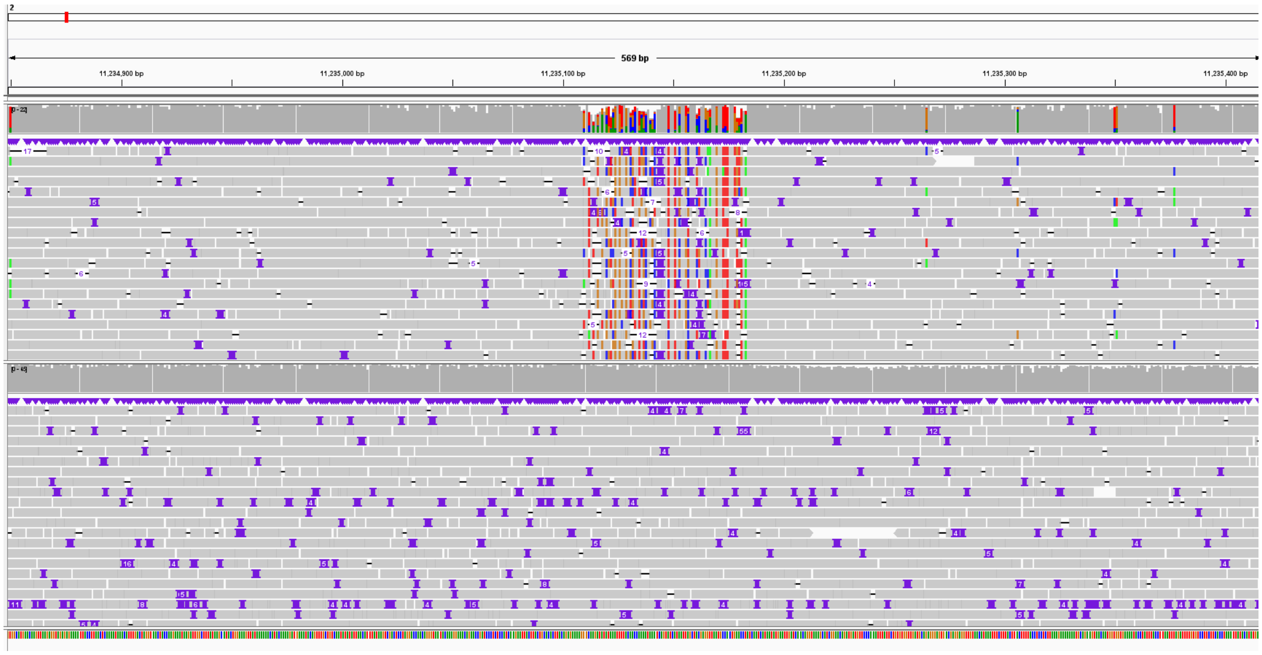

Figure S26 | CSUB chromosome 2, position 11235109, length 74 bp

Figure 27 & 28: This SV is a confirmed insertion, as can be seen in the overall alignment and the individual PacBio alignments.

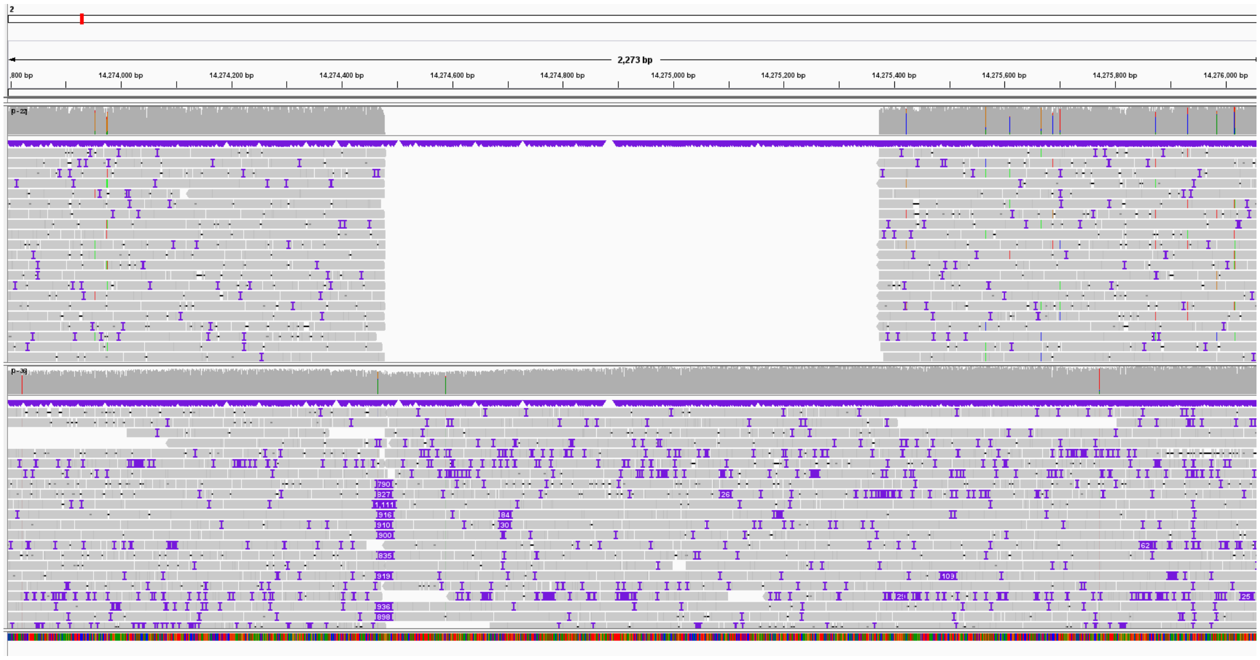

Figure S27 | CSUB chromosome 2, position 14274478, length 895 bp.

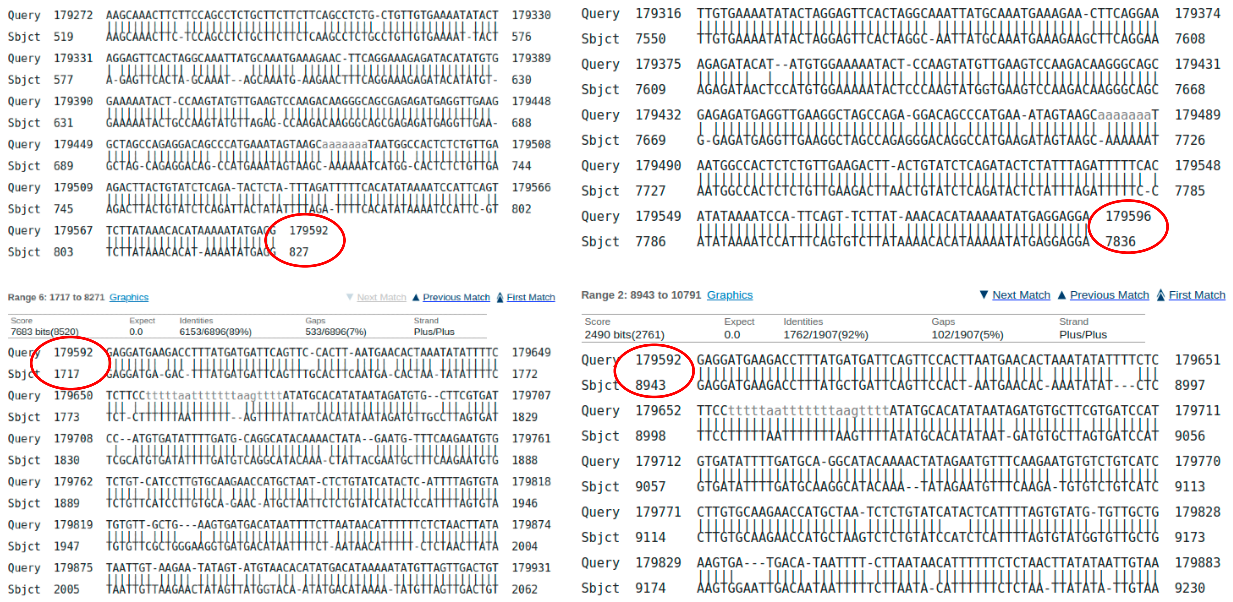

Figure S28 | Individual alignment of two PacBio reads for ‘CSUB chromosome 2, position 14274478, length 895 bp’.

Figure 29 : We did not observe a SV at this position by inspecting the alignments.

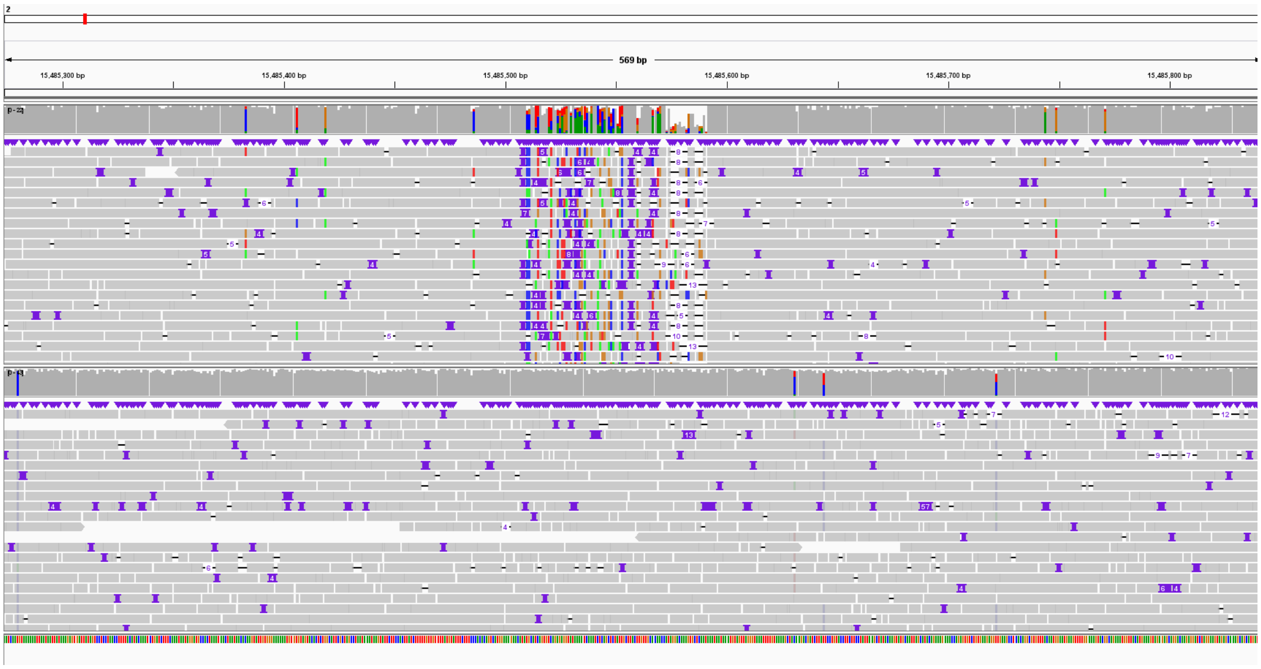

Figure S29 | CSUB chromosome 2, position 15485508, length 895 bp.

Figure 30 & 31: Insertions were observed across a tandem repeat region.

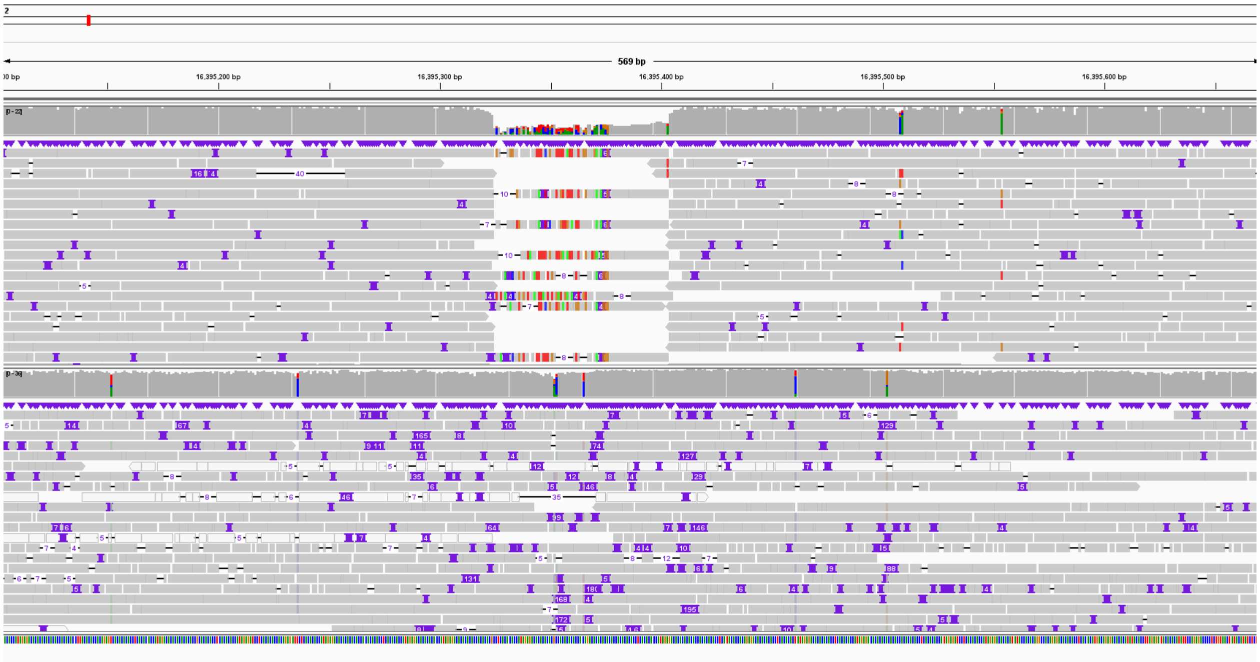

Figure S30 | CSUB chromosome 2, position 16395324, length 54 bp.

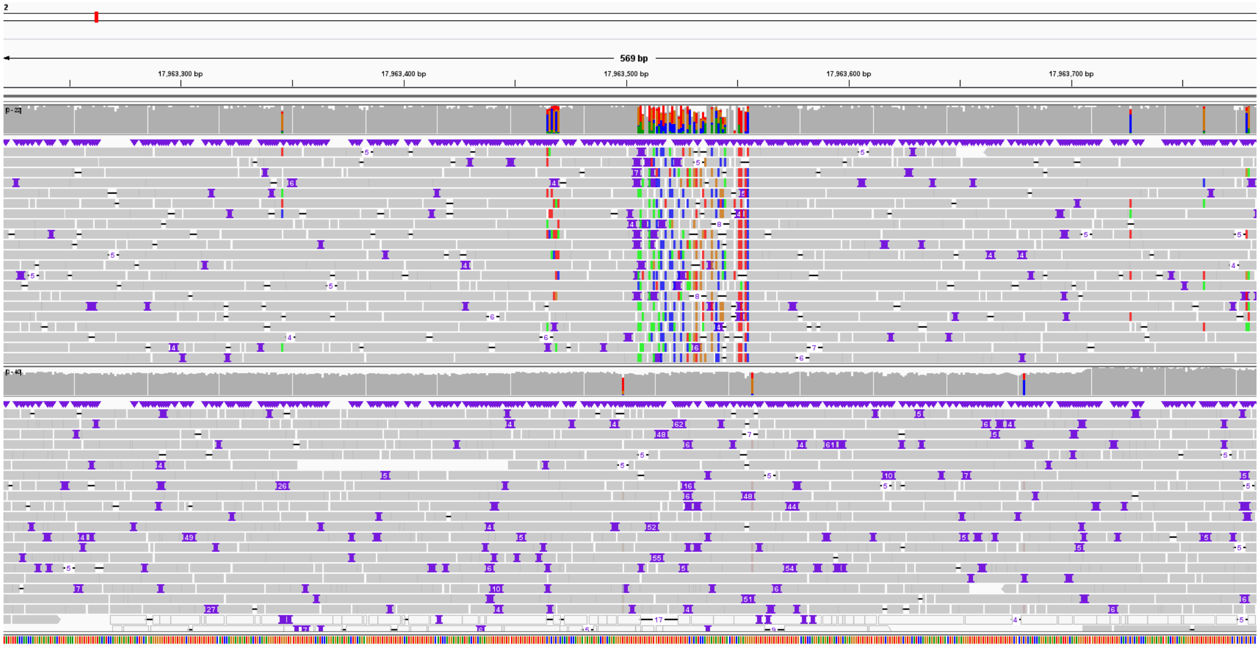

Figure S31 | CSUB chromosome 2, position 17963505, length 50 bp.

Figure 32: Insertions were observed across a repetitive region.

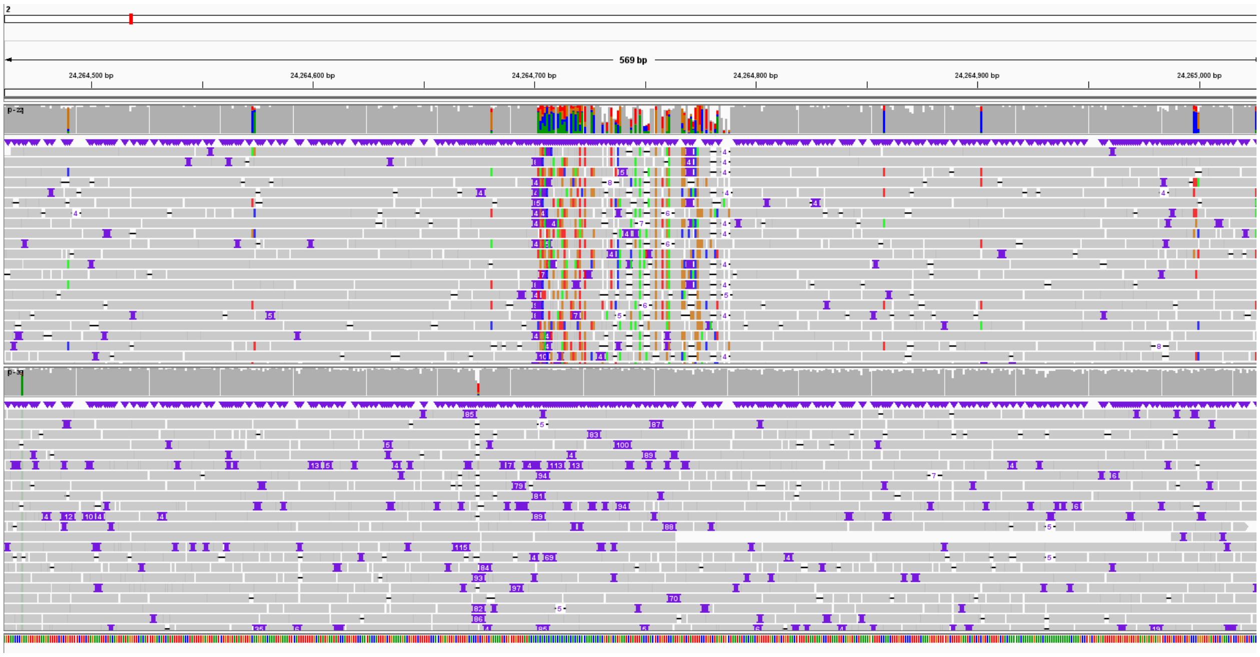

Figure S32 | CSUB chromosome 2, position 24264701, length 87 bp.

Figure 33 & 34 : We did not observe a SV at either position by inspecting the alignments.

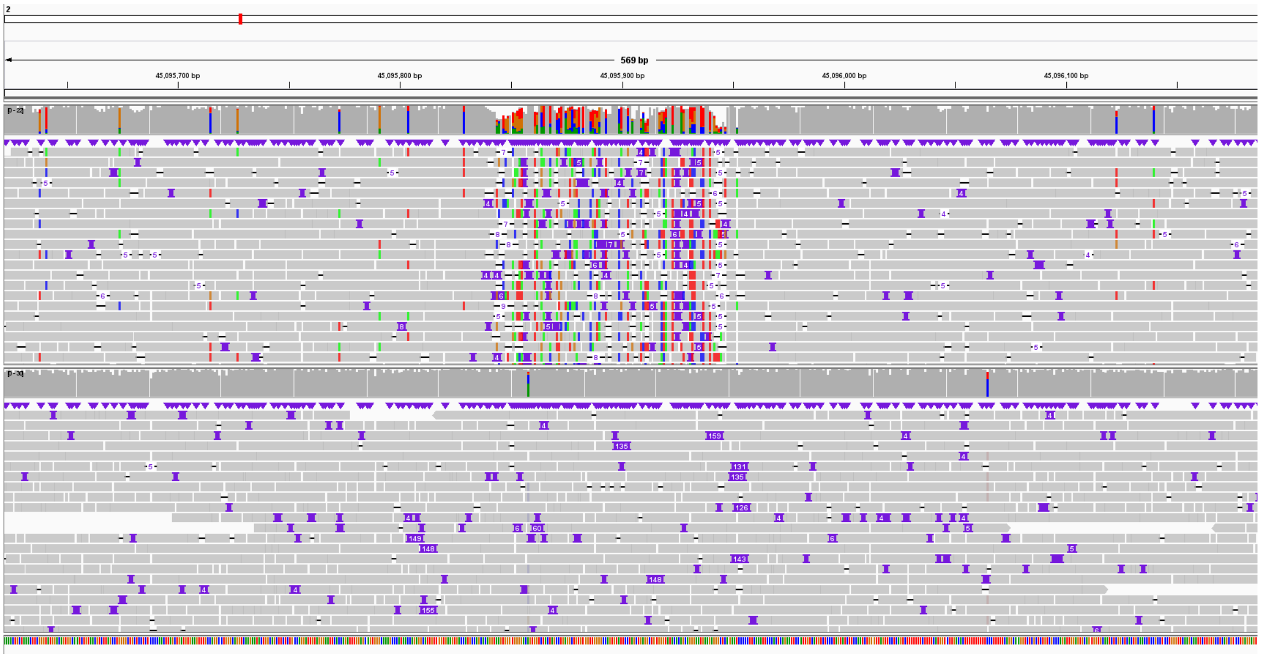

Figure S33 | CSUB chromosome 2, position 45095843, length 110 bp.

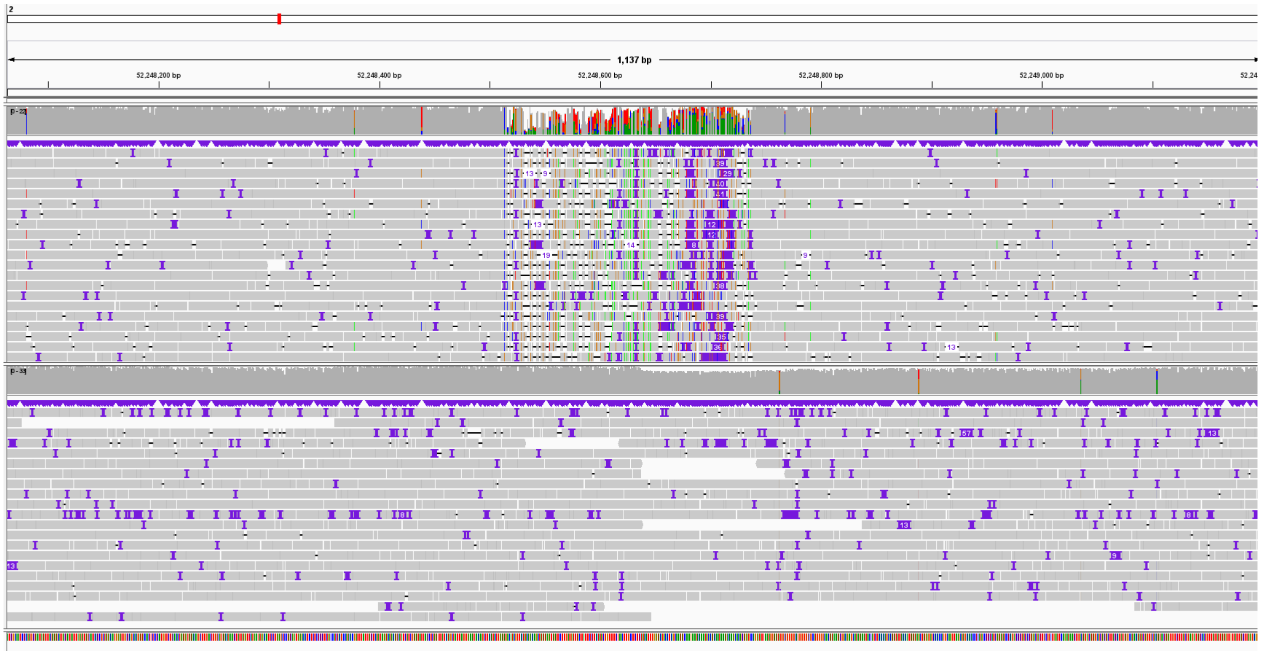

Figure S34 | CSUB chromosome 2, position 52248513, length 225 bp.

Figure 35 & 36: Insertions were observed across a repetitive region.

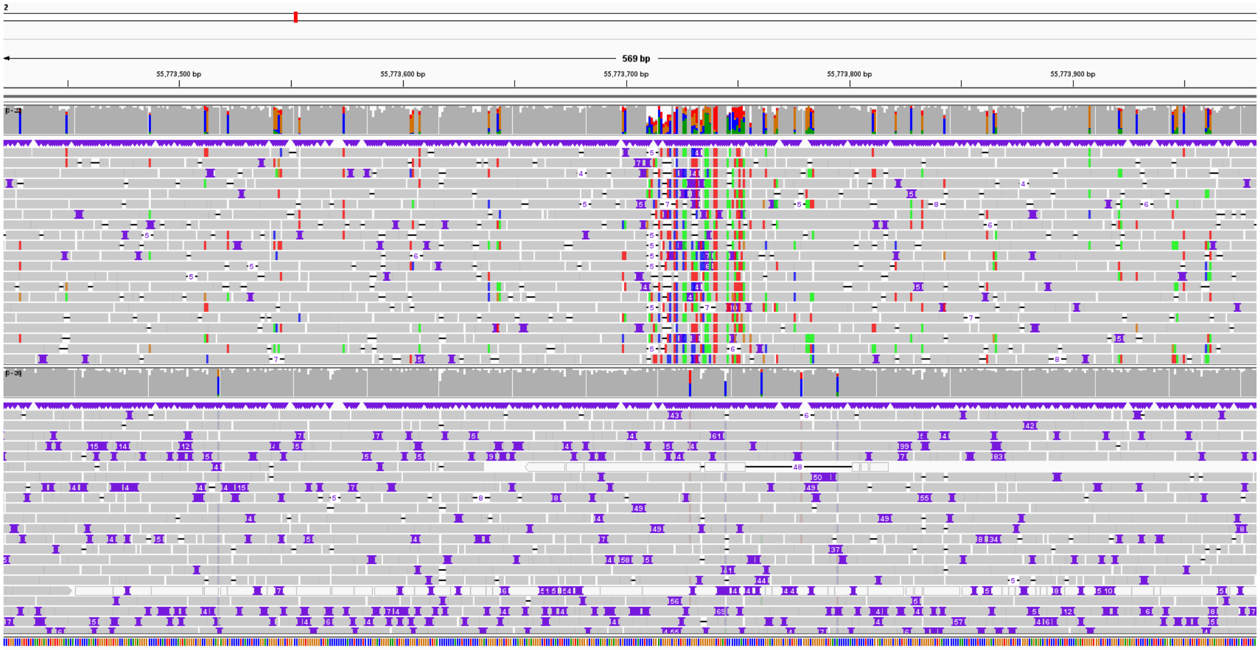

Figure S35 | CSUB chromosome 2, position 55773706, length 50 bp.

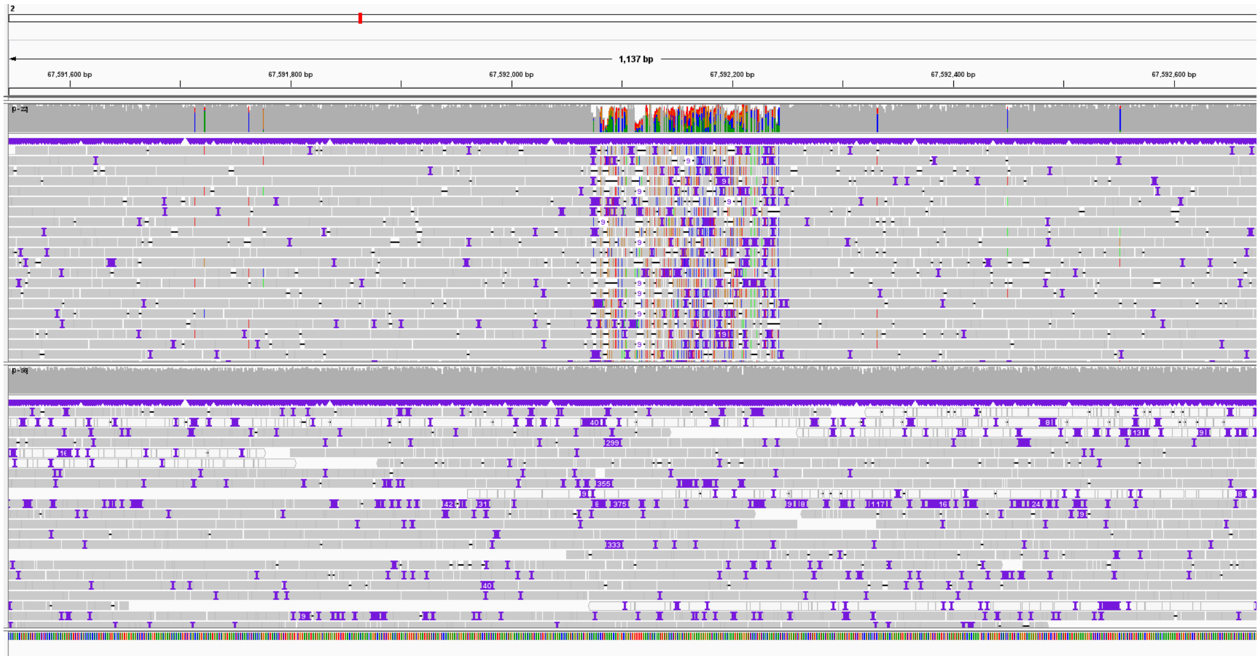

Figure S36 | CSUB chromosome 2, position 67592074, length 169 bp.

Figure 37: We observed an heterozygous deletion.

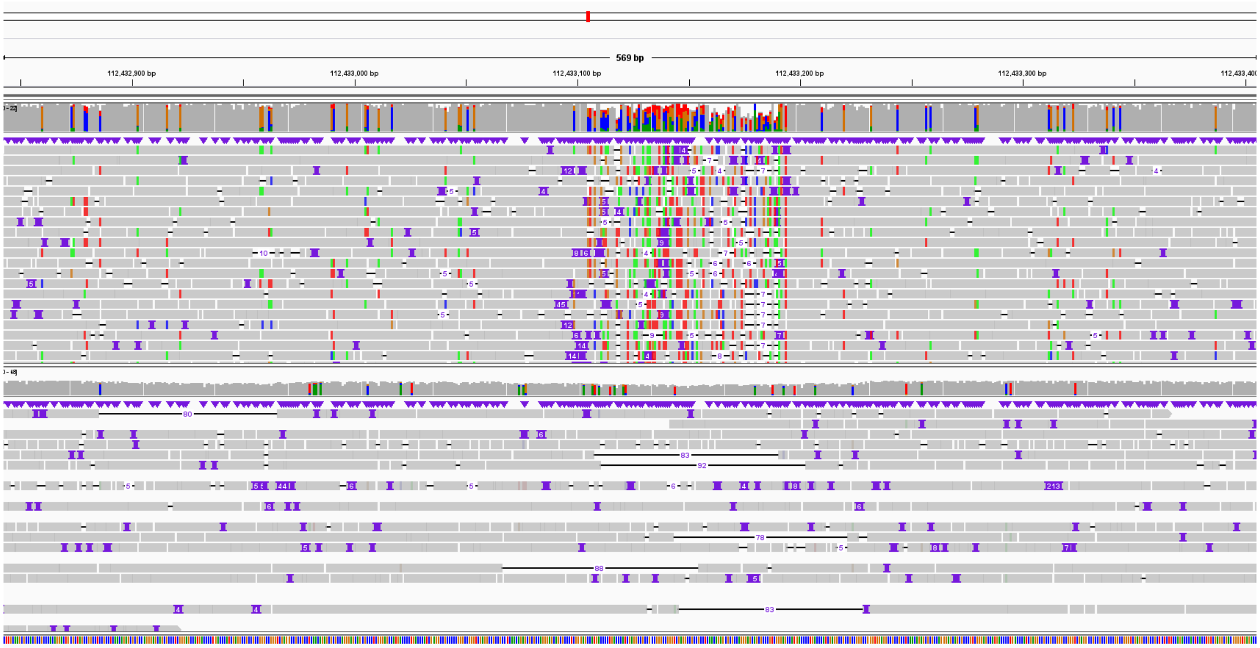

Figure S37 | CSUB chromosome 2, position 112433104, length 90 bp.

Figure 38: Insertions were observed across a tandem repeat region.

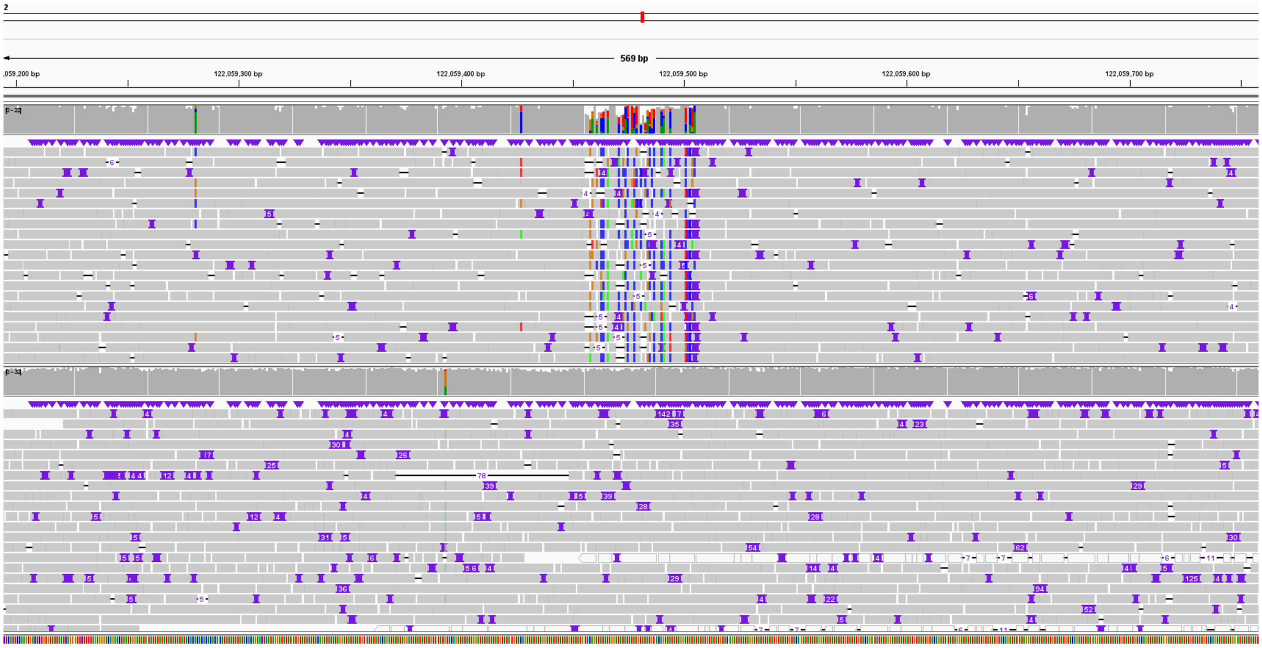

Figure S38 | CSUB chromosome 2, position 122059455, length 50 bp.

References

1 . Chaisson, M.J.P., et al. (2019). Multi-platform discovery of haplotype resolved structural variation in human genomes. *Nature Communications*, **10**, 1784. doi.org/10.1038/s41467-018-08148-z
