# Supplementary material for: A benchmark of structural variation detection by long reads through a realistic simulated model
Source: Genome Biol. 2021 Dec 15;22:342. doi: 10.1186/s13059-021-02551-4 (PMC8672642; doi:10.1186/s13059-021-02551-4)
Supplement: Supplementary file 3 — Additional file 3 Review history. [file 13059_2021_2551_MOESM3_ESM.docx]

Review History

**First round of review**

**Reviewer 1**

**Were you able to assess all statistics in the manuscript, including the appropriateness of statistical tests used?**

There are no statistics in the manuscript.

**Comments to author:**

In the manuscript "A benchmark of structural variation detection by long reads through a realistic simulated model", Dierckxsens et al. described a simulator, named Sim-it, to generate long read data with predesigned or randomly generated SVs, and an ensemble SV discovery method, named combiSV to integrate and filter SV calls from different methods. Both methods are useful for genomic variants discoveries, but more details of the methods and benchmarking results should be provided to improve clarity of the manuscript. Here are my comments / suggestions:

1. One strength of Sim-it versus other simulators is the customized error profiles that were trained and implemented in Sim-it. However, it is not clearly stated in the manuscript how these profiles were trained. Information regarding the computational methods used to generate the error profiles, such as the machine learning model (if applied) and corresponding parameters, should be included. In addition, exact information of the sample adopted to train the error profile for PromethION should be provided.

2. In figure2, a 'Total Score' is calculated for each SV discovery method, but the method for the calculation is not explained. Can the authors describe how the recall and precision are combined to calculate the 'Total Score'?

3. Correlation between sequencing length and SV discovery power is also discussed in the manuscript (last paragraph under "Structural variance detection using simulated long reads"), and the results are interesting. Can the authors provide a figure like Figure 2 or a table for the analyses to help clarify the results?

4. The authors pointed that performance of the algorithms on real data is much worse than simulated data, and this might reflect low accuracy of the simulator. Adding a discussion about the potential causes of the difference would be useful. In addition, results of the real data were interesting, but the description were not clear enough. Adding a figure or table for the analyses would help.

5. The authors compared the strengths and weaknesses of different SV algorithms, and implemented these information into combiSV. I think the method design is smart, but the method is not described with enough detail. For example, What are the strength of each algorithm? And what are the weakness? How is the model trained?

6. The figures seem to be off from the manuscript and the figure legends.In the manuscript, Figure 1-3 were cited but only 2 figures were included; In addition, legend for figure 1-5 were provided with the manuscript, but they do not seem to match the actual figure that:

1. description of subpanels in figure 1 were not provided in the corresponding legend

2. there are no subpanels in figure 2 but descriptions of 4 subpanels were included in the legend

3. figure 3-5 were not included

**Reviewer 2**

**Were you able to assess all statistics in the manuscript, including the appropriateness of statistical tests used?**

Yes, and I have assessed the statistics in my report.

**Comments to author:**

In this works, the authors have developed a new method for simulating SV using long-read sequencing data. Furthermore, they report an ensemble approach for combining SVs from multiple callers. They have tried to compare their simulator with other long-read simulators.

Overall, despite being a useful tool, it's unclear how useful Sim-it is compared to other simulators in the current manuscript.

Major comments:

1. The rationale for developing a simulator was described as due to a lack of gold standard SVs. However, in the current study, they use Chaisson et al. as the gold standard. This isn't very clear. Authors should explain why some of the long-read-based SV resources can't be used for benchmarking SVs?

2. Overall, for various figures, legends are not very helpful. What does color code mean in Figure1? What does count refer to in panels C & D of Figure1?

3. The author should provide some form of quantitative measurement to show that their method performs better than others in capturing error profiles.

4. Sniffle seems to perform well for the majority of datasets except for Pacbio-HiFi. This is surprising as one would expect that better chemistry of HiFi data should be better for calling SVs. Can authors provide a rationale for this inconsistency?

5. Also, why FP rate increases with sequencing depth above 30X?

6. The authors didn't provide any relevant details in the method or elsewhere about how combiSV works.

7. Authors should compare combiSV with other ensemble approaches like survivor, parliament & fusor-SV.

**Reviewer 3**

**Were you able to assess all statistics in the manuscript, including the appropriateness of statistical tests used?**

Yes, and I have assessed the statistics in my report.

**Comments to author:**

The authors of the manuscript "A benchmark of structural variation detection by long reads through a realistic simulated model." Describe their method to simulate reads and Structural Variations to improve the method development and testing of SV Callers. They further describe a new merging of SV method combiSV that allows the comparison of SV of a predefined set of SV VCF files. The manuscript is well written and easy to follow. The figures however are barely readable including the table and some captions.

In the following I list my questions and concerns:

1. I don't quite understand how this is different to SURVIVOR (https://github.com/fritzsedlazeck/SURVIVOR) that was published 4 years ago. To be precise: it includes SV simulation (all different types including 2 complex chained SVTypes), an read simulator that derives read distribution and error profile from a provided bam file if the user wants and allows the comparison of SV across many VCF files. SURVIVOR also simulates SNV to have a more complex scenario for mappers.

I acknowledge that SURVIVOR does just randomly place the SV across the genome vs. here its more of a centromeric/ telomeric pattern.

2. You should better explain what a complex substitution looks like? I assume a insertion and deletion on the same spot ? How would a SV caller report this vs. an SNV caller ? I am aware how an inverted duplication looks like but still this is not standard and should be further explained.

3. I think you should compare more to other read simulators. Like what makes your better or why one should use yours?

4. Same for other SV simulation methods.

5. I think you confuse haplotype with genotype.

6. What are the evaluation criteria for your method? Can this be user defined ? Are you providing a method for that? Your method section does not say what tool you used.

7. Your documentation on your Github points to your wiki which has 2-3 lines written.

8. The figure resolution is really bad.

9. Why do the Deletions and Insertions show in the figures a size of 1-8bp? SV start typically at 50bp .

10. CombiSV seems tailored to the here specific SV Callers? Did you test different parameters to see what thresholds works best ?

**Authors Response**

**Point-by-point responses to the reviewers’ comments:**

1.	One strength of Sim-it versus other simulators is the customized error profiles that were trained and implemented in Sim-it. However, it is not clearly stated in the manuscript how these profiles were trained. Information regarding the computational methods used to generate the error profiles, such as the machine learning model (if applied) and corresponding parameters, should be included. In addition, exact information of the sample adopted to train the error profile for PromethION should be provided.

*Per the suggestion, we added more information about the customized error profiles under the section “Train customized error profiles for Sim-it”. We used a publicly available dataset for the PromethION error profile, we added this information to the paragraph.*

2.	In figure2, a 'Total Score' is calculated for each SV discovery method, but the method for the calculation is not explained. Can the authors describe how the recall and precision are combined to calculate the 'Total Score'?

*The total score is explained under the section “SV detection on simulated reads” from Materials and Methods:*

*“As there are multiple metrics that define the performance of an SV detection algorithm, we adopted an overall score that that combines each of the metrics. For each detected SV, a maximal score of 1 was possible; 0.4 for the correct position, 0.2 for the correct length, 0.2 for the correct type of SV and 0.2 for the correct genotype. The scores for length and position proportionally decreased with difference compared to the reference set. Finally, the number of false positives were subtracted from the total score and eventually expressed as a percentage of the maximum possible score”*

3.	Correlation between sequencing length and SV discovery power is also discussed in the manuscript (last paragraph under "Structural variance detection using simulated long reads"), and the results are interesting. Can the authors provide a figure like Figure 2 or a table for the analyses to help clarify the results?

*We added an additional figure and included two additional data points (read lengths of 75k and 100k).*

4.	The authors pointed that performance of the algorithms on real data is much worse than simulated data, and this might reflect low accuracy of the simulator. Adding a discussion about the potential causes of the difference would be useful. In addition, results of the real data were interesting, but the description were not clear enough. Adding a figure or table for the analyses would help.

*Our simulated datasets were based on the largest SV study to date (Chaisson et al). If we would use the detected SVs (with their exact position, genotype and type of SV) of this study as a gold standard to score the different SV algorithms on a real long read dataset of this study, the results were much worse than the simulated datasets. But the cause of this is the inaccurate calling of SVs in the Chaisson et al study (and probably most studies) and not because of an inaccurate simulation. In the supplementary materials, we also showed the incorrect calling of complex substitutions in the Chaisson et al study.*

*To point this further out we added an additional simulation and benchmark of the GIAB SV callset. This SV callset, which is carefully manually curated, is probably one of the most accurate SV callsets to date (but is limited to deletions and insertions and does not cover the complete human genome). SV calling on the simulation of this dataset gave similar results to the real data.*

5.	The authors compared the strengths and weaknesses of different SV algorithms, and implemented these information into combiSV. I think the method design is smart, but the method is not described with enough detail. For example, What are the strength of each algorithm? And what are the weakness? How is the model trained?

*We described the method more thoroughly under the section “combiSV” of Materials and Methods. The code is also publicly available and can be looked at on github. The strengths and weaknesses of each algorithm are discussed under the section ‘Discussion’.*

6.	The figures seem to be off from the manuscript and the figure legends.In the manuscript, Figure 1-3 were cited but only 2 figures were included; In addition, legend for figure 1-5 were provided with the manuscript, but they do not seem to match the actual figure that:

*We also noticed that the figures were almost unreadable and had the wrong legends. We contacted the editorial office, apparently there was an issue with their submission system in the latex format. I hope this time the problem will be resolved, otherwise I think the figures can be downloaded individually. Also, there were only 2 figures and 3 tables, this seems to mess up the legend order.*

Revisions reviewer 2

1.	The rationale for developing a simulator was described as due to a lack of gold standard SVs. However, in the current study, they use Chaisson et al. as the gold standard. This isn't very clear. Authors should explain why some of the long-read-based SV resources can't be used for benchmarking SVs?

*We have used the Chaisson et al study as a reference for the simulation as it is the largest SV study to date. We are using it as a gold standard for the amount of SVs present in the human genome. Although it cannot be used as a gold standard to compare tools with real data, as the the calls are still inaccurate. This is made clear by the incorrectly called complex substitutions and the large discrepancy between the SV detection results of the real data of the Chaisson et al study and the simulated data based on this study. The GIAB dataset is the only true golden standard in accuracy (but incomplete), a simulation of this dataset has similar results as the real data. We added this comparison and additional simulation under section ‘Structural variance detection using real datasets’.*

2.	Overall, for various figures, legends are not very helpful. What does color code mean in Figure1? What does count refer to in panels C & D of Figure1?

*We also noticed that the figures were almost unreadable and had the wrong legends. We contacted the editorial office, apparently there was an issue with their submission system in the latex format. I hope this time the problem will be resolved, otherwise I think the figures can be downloaded individually. Also, there were only 2 figures and 3 tables, this seems to mess up the legend order.*

3.	The author should provide some form of quantitative measurement to show that their method performs better than others in capturing error profiles.

*The figures were in very low quality as explained above. Figure 1 shows heatmaps of the error profiles of Sim-t compared to the real error profiles. We also made heatmaps of the other tools, they can be found in supplementary materials. Sim-it is not the only simulator with an accurate error profile, LongISLND and Badread also had similar error profiles compared to the real data. Besides the heatmap, we looked at the length of erroneous deletions and insertions (panel C and D of figure 1). These figures show that for example Badread has too many deletions and insertions (can also be seen on the heatmap) compared to mismatches and that LongISLND has proportionally to many short deletions of 1 bp.*

4.	Sniffles seems to perform well for the majority of datasets except for Pacbio-HiFi. This is surprising as one would expect that better chemistry of HiFi data should be better for calling SVs. Can authors provide a rationale for this inconsistency?

*Sniffles performs indeed a bit less for the HiFi dataset, this is due to a lower recall compared to the other tools. Sniffles seem to have a larger drop in recall with shorter read lengths, hence the HiFi dataset has a median length of 15 kbp, while the Nanopore and PacBio long reads have a median length of 25 kbp. Figure 3 is a new figure that shows the impact of the read length, Sniffles shows a large drop in recall for the 15 kbp Nanopore reads. We would like to also note that we used minimap2 as an aligner for Sniffles, while they recommend NGMLR and therefore it could be that Sniffles does not perform optimally. We choose for minimap2 as this is by far the most popular and user friendly aligner for long reads.*

*Although PacBio has a lower recall, the high accuracy of the reads increases the precision and percentage of perfect matches for all datasets. The strong improvement in perfect matches is thanks to more accurate position and length calls.*

5.	Also, why FP rate increases with sequencing depth above 30X?

*The drop in precision at 20x and 50x is explained by the increasing sequencing depth vs. the minimum read support number. A lower minimum read support will increase the recall but reduce the precision and vice versa, it is therefore necessary to select a number that gives a good balance between recall and precision. We choose a minimum read support number of 3 for the 10x and the 20x datasets and 5 for the 30x and the 50x datasets. Another option would be to filter based on the support ratio*

6.	The authors didn't provide any relevant details in the method or elsewhere about how combiSV works.

*Thanks for the suggestion. We described the method more thoroughly under the section “combiSV” of Materials and Methods . The code is also publicly available and can be looked at on github.*

7.	Authors should compare combiSV with other ensemble approaches like survivor, parliament & fusor-SV.

*We added SURVIVOR to the benchmark study, although this tool only combines VCF files by overlap and not by individual strengths of the SV callers. Parliament and fusor-SV are both tools for short sequencing reads and are not suited for long read SV callers and were therefore not included to the benchmark.*

Revisions reviewer 3

1.	I don't quite understand how this is different to SURVIVOR

(https://github.com/fritzsedlazeck/SURVIVOR) that was published 4 years ago. To be precise: it includes SV simulation (all different types including 2 complex chained SVTypes), an read simulator that derives read distribution and error profile from a provided bam file if the user wants and allows the comparison of SV across many VCF files. SURVIVOR also simulates SNV to have a more complex scenario for mappers. I acknowledge that SURVIVOR does just randomly place the SV across the genome vs. here its more of a centromeric/ telomeric pattern.

*Thanks for the suggestion and we added it to our benchmark. SURVIVOR is indeed a similar versatile tool, although there are significant differences which made it unsuitable for this study. For the combination of VCFs, SURVIVOR only combines VCF files based on overlap and does not look at the individual strengths of the SV callers. This makes SURIVOR more limited and the results can vary strongly depending on which tools are combined. It even depends on which order you give in the tools. It often does not perform better than a single existing tool and always has a lower recall and precision then combiSV. combiSV performs consistently better than the existing tools for any combination of 3 tools (or all 6 tools), as long as cuteSV or Sniffles is included.*

*We also tested the SV simulation option, but this one is also limited. It does not support complex substitutions, it cannot provide realistic distribution of SVs, it cannot insert foreign sequences and does not support more than one haplotype. Furthermore, it is not possible to simulate 24,000 SVs, there is a maximum amount. In the github they mention that it is not designed to simulate a large amount of SVs. We do not know the exact limit but 24,000 did not work. It does indeed also simulate SNVs, but we will also add this to the next update of Sim-it.*

*We made heatmaps of the error profiles they provided and from error profiles we created with their tool, all heatmaps showed a very simplified error profile. Another important shortcoming is that you can not adjust the read lengths, instead it will take over the read length distribution from the trained error profile. We also added a sequencing depth profile option to Sim-it.*

2.	Overall, You should better explain what a complex substitution looks like? I assume a insertion and deletion on the same spot ? How would a SV caller report this vs. an SNV caller ? I am aware how an inverted duplication looks like but still this is not standard and should be further explained.

*It is indeed a deletion and an insertion at the same location. It could also be seen as a small translocation, a term often used for very large SVs. I am not sure how a SNV caller would report this, it could be seen as many SNVs, although alignments will be often of low quality in that region, which would lead to removal of these SNVs in the filter step. We were surprised of the high amount of detected complex substitutions in the Chaisson et al. study, but you can see in the supplementary materials, most of them were incorrectly interpreted (probably caused by conflicting SV calls of different methods). So the exact amount of complex substitutions is probably lower than in that study, nevertheless it is hard to know as none of the current SV callers was able to detect many of the simulated complex substitutions in our study. We explained both complex substitutions and inverted duplications in the section ‘Structural variation simulation benchmark’.*

3.	I think you should compare more to other read simulators. Like what makes your better or why one should use yours?

*I know the figures were in very low quality, but Figure 1 shows heatmaps of the error profiles of Sim-t compared to the real error profiles. We also made heatmaps of the other tools, they can be found in supplementary materials. In supplementary tables we also compare the features and system requirements. Sim-it is not the only simulator with an accurate error profile, LongISLND and Badread also had similar error profiles compared to the real data. Besides the heatmap, we looked at the length of erroneous deletions and insertions (panel C and D of figure 1). These figures show that for example Badread has too many deletions and insertions (can also be seen on the heatmap) compared to mismatches and that LongISLND has proportionally to many short deletions of 1 bp. You pointed us out about SURVIVOR, which should have been included so added this to the comparison. And we know Sim-it is not a big advancement in the field, but we believe that the complete benchmark study and combiSV will be useful for many SV studies.*

4.	Same for other SV simulation methods.

*We compared the SV simulators in table 1. We wanted to give an overview what was possible with each simulator and compare the system requirements. We developed Sim-it to design the SV calling benchmark and to create combiSV. We see the results of the benchmark and combiSV as the main contribution to the field from our study. Sim-it was more a tool to achieve this and can we think table 1 shows the strengths of Sim-it, but also the strengths of other tools.*

5.	Sniffles seems to perform well for the majority of datasets except for Pacbio-HiFi. This is surprising as one would expect that better chemistry of HiFi data should be better for calling SVs. Can authors provide a rationale for this inconsistency?

*Sniffles performs indeed a bit less for the HiFi dataset, this due to a lower recall compared to the other tools. Sniffles seem to have a larger drop in recall with shorter read lengths, hence the HiFi dataset has a median length of 15 kbp, while the Nanopore and PacBio long reads have a median length of 25 kbp. Figure 3 is a new figure that shows the impact of the read length, Sniffles shows a large drop in recall for the 15 kbp Nanopore reads. We would like to also note that we used minimap2 as an aligner for Sniffles, while they recommend NGMLR and therefore it could be that Sniffles does not perform optimally. We choose for minimap2 as this is by far the most popular and user-friendly aligner for long reads.*

6.	I think you confuse haplotype with genotype.

*Thanks and we made the changes.*

7.	What are the evaluation criteria for your method? Can this be user defined ? Are you providing a method for that? Your method section does not say what tool you used.

*Because existing evaluation tools are limited to measuring recall and precision values, we opted to use a customized script to evaluate the SV calls. Our script does not just measures the recall and precision, but it also determines how accurate the position, type, length and genotype are called. Plus additional metrics for each type of SV. We were recently contacted to make our comparison scripts available to copy our study for another species. Therefore we will add the comparison scripts to the sim-it github page.*

8.	Your documentation on your Github points to your wiki which has 2-3 lines written.

*On the side panel of the wiki page, you have two subtopics for long read and SV simulation. I will add a link to both in the main panel too.*

9.	The figure resolution is really bad.

*We also noticed that the figures were almost unreadable and had the wrong legends. We contacted the editorial office, apparently there was an issue with their submission system in the latex format. I hope this time the problem will be resolved, otherwise I think the figures can be downloaded individually. Also, there were only 2 figures and 3 tables, this seems to mess up the legend order.*

10.	Why do the Deletions and Insertions show in the figures a size of 1-8bp? SV start typically at 50bp .

*Because the figures are barely readable, it is indeed not clear. But those are the lengths of erroneous deletions and insertions of the simulated Nanopore reads. The length of these errors are important as larger deletions or insertions are more problematic for SV calling.*

11.	CombiSV seems tailored to the here specific SV Callers? Did you test different parameters to see what thresholds works best ?

*combiSV worked indeed only on these 5 SV callers, but we will add new tools if there are any. We added for example cuteSV, which we also added to the benchmark in the revisions. As for the parameters, combiSV has only one adjustable parameter and that is the ‘minimum read support’. We tested this parameter for all SV callers to see which value gave overall the best performance. But combiSV uses the unfiltered VCF files of the different SV callers, so the default values suffice.*

**Second round of review**

**Reviewer 1**

Reviewer #1: Major:

1. My biggest concern of this manuscript lies in its novelty. I think both Sim-it and combiSV are valuable computational tools, however, there are not enough novel biological findings revealed in this manuscript. Thus, a method-focused journal would be better suited for this manuscript.

2. The description of accuracy of Sim-it versus other simulators is not clear enough. The authors showed the error pattern of simulated and real data in heatmap, but did not provide any quantitative statistics in the paragraph (line 47-62) for direct comparison of the accuracy. Thus it's hard to interpret the real performance of Sim-it than other simulators.

3. When looking at "Total Score" in table 2, combiSV showed significantly increased performance than individual SV algorithms. However, when calculating F-score, which is the commonly adopted statistics to integrate precision and recall, the superiority is not as significant (for example, combiSV(3) has an F-score of 88.5% for nanopore data, while F-score for sniffles is 87.8% for the same data). To fairly represent the results, I suggest adding F-score to table 2. In addition, the increased performance of combiSV were built on the results from 6 SV callers , thus computational efficiency of 6 SV callers versus individual SV algorithm should be compared for comprehensive representation of this tool.

4. Significant improvement is required to further polish this manuscript. There are statements throughout the manuscript that could be improved for clarity (see minor comments), and multiple places where proper citations need to be added. In addition, detailed legend for the figures need to be added.

5. Table 3 is not provided except for the legend

Minor:

1. None of the supplemental figures or tables were explicitly cited in the manuscript, instead they were all referred to as "Supplementary Data". This makes it hard to find the correct supportive data for each statement, and I suggest adding the exact number of supplemental figure /table to the places where they were referred to and ensure every single table / figure in the supplementary material is cited.

2. "SV" is not defined before used in the abstract;

3. The author mentioned "We assessed the performance of 7 long read SV detection algorithms through a realistic model of 24,600 SV events. " but did not provide any information of the SVs before describing the results. The source and a brief description of the SVs should be provided here, or the corresponding method section should be cited.

4. I suggest adding a full list of the 9 simulators and 7 long-read SV algorithms with their correspondence publications at the beginning of their section so it's easier for audiences to understand the results.

5. There are multiple grammar errors throughout the manuscript, and I suggest a systematic revision of the grammar. In addition, proper citations should be added to several statements.

1. "which makes these technologies (PacBio and Oxford Nanopore) currently the best option for structural variance detection [8].".

a. Full name of "PacBio" should be explained before the abbreviation is used;

b. Signature papers from PacBio and Oxford Nanopore should be cited respectively

c. It should be "options" instead of "option"

d. "ONT" need to be defined here before it's used in the rest part of the manuscript

2. "The downside of these longer reads are their lower accuracies (85-95%) compared to NGS reads (> 99%)" Source of the numbers should be cited

3. "Varsim can generate long reads through PBSIM or LongISLND." All 3 algorithms should be cited. Same for all other algorithms mentioned in the manuscript.

**Reviewer 2**

The authors have tried to address some of the concerns that I raised earlier in the updated manuscript. However, they still need to provide more detail about their method and clarify the precision-recall scores reported in the table2. Please see specific comments below.

The authors have tried to address my earlier concern by adding the "combiSV" sub-section under the Method section. Unfortunately, this sub-section still doesn't provide enough detail. They should extend this sub-section further and provide more detail, particularly related to the equation mentioned in the added text.

Can the authors clarify whether the precision and recalls score of SURVIVOR reported in Table2 is the highest score for any combination of 3 SV tools used in the SURVIVOR method?

**Authors Response**

**Point-by-point responses to the reviewers’ comments:**

Revisions reviewer 1

1. My biggest concern of this manuscript lies in its novelty. I think both Sim-it and combiSV are valuable computational tools, however, there are not enough novel biological findings revealed in this manuscript. Thus, a method-focused journal would be better suited for this manuscript.

*It will be considered as a “Method” article, so the editor confirmed this is not an issue.*

2. The description of accuracy of Sim-it versus other simulators is not clear enough. The authors showed the error pattern of simulated and real data in heatmap, but did not provide any quantitative statistics in the paragraph (line 47-62) for direct comparison of the accuracy. Thus it's hard to interpret the real performance of Sim-it than other simulators.

*We opted for showing heatmaps as visualization because we thought it gives an intuitive representation of the error profile. But behind each square there are quantitative values. Overall deletion, insertion and mismatch values are easy to simulate and are too limited to define the accuracy of an error profile, therefore we looked at each of these values for all nucleotide patterns. This reveals systemic error patterns and are important to simulate as random distribution of errors would improve SV calling compared to real data. But to add additional qualitive values, we added three tables to the supplementary tables. Table 2 and 3 of supplementary tables Show general statistics of the simulations, while Table 4 compares the Euclidean distance of the context-specific error patterns and the distributions for the lengths of deletions and insertions. These numbers give similar results as the heatmaps and the graphs, with only LongISLD better than Sim-it in the context-specific error patterns and Sim-it as the most accurate in insertion and deletion length distributions. Sim-it and LongISLD are the most accurate read simulators, with each their strengths. We think that shorter lengths in deletions will affect mapping quality in a more profound way than less accurate context-specific error patterns of single positions. But for most purposes both simulators would work well enough.*

3. When looking at "Total Score" in table 2, combiSV showed significantly increased performance than individual SV algorithms. However, when calculating F-score, which is the commonly adopted statistics to integrate precision and recall, the superiority is not as significant (for example, combiSV(3) has an F-score of 88.5% for nanopore data, while F-score for sniffles is 87.8% for the same data). To fairly represent the results, I suggest adding F-score to table 2. In addition, the increased performance of combiSV were built on the results from 6 SV callers , thus computational efficiency of 6 SV callers versus individual SV algorithm should be compared for comprehensive representation of this tool.

*We also added the F-score to the table. The total score shows indeed larger differences than you can see in precision and recall percentages. The reason why we added the total score is because precision and recall don’t show the complete picture and can vary greatly depending on how tolerant you match SV calls to the reference set. We chose a tolerant matching algorithm, were we match SV calls independently from type, genotype, length and within a range of 1600 bp. As precision and recall metrics assess SV calls as 1 or 0, we wanted a score that includes accuracy of the calls, which is important for SV studies, but not represented in the F-score. The downside of our total score is that some SV callers that do not call SV types or genotypes are too heavily punished. Currently, SURVIVOR is a frequently used tool to build a consensus, while the output has sometimes a lower F-score than one of the 3 combined tools. Therefore we wanted to create an ensemble tool, that will always create an improved SV call set. Besides an improved F-score, it will also generate more accurate calls.*

*As for the computational efficiency, we added a benchmark for the system requirements. Although the alignment phase usually consumes most of the wall time and memory consumption, some SV callers would have an impact. We added a table in SI and referred to it in the main text.*

4. Significant improvement is required to further polish this manuscript. There are statements throughout the manuscript that could be improved for clarity (see minor comments), and multiple places where proper citations need to be added. In addition, detailed legend for the figures need to be added.

*We did an additional grammar check and addressed the minor comments and included citations.*

1. None of the supplemental figures or tables were explicitly cited in the manuscript, instead they were all referred to as "Supplementary Data". This makes it hard to find the correct supportive data for each statement, and I suggest adding the exact number of supplemental figure /table to the places where they were referred to and ensure every single table / figure in the supplementary material is cited.

*Referred to all the figures of Supplementary Materials*

2. "SV" is not defined before used in the abstract;

*Defined it in the abstract*

3. The author mentioned "We assessed the performance of 7 long read SV detection algorithms through a realistic model of 24,600 SV events. " but did not provide any information of the SVs before describing the results. The source and a brief description of the SVs should be provided here, or the corresponding method section should be cited.

*We extended the description in the methods section and referred to that section*

4. I suggest adding a full list of the 9 simulators and 7 long-read SV algorithms with their correspondence publications at the beginning of their section so it's easier for audiences to understand the results.

*There were already lists with their citations in the corresponding methods section. Since Genome biology has the methods section in the end, it is indeed after the other paragraphs, so now we referred specifically to those methods sections.*

5. Table 3 is not provided except for the legend

*Not sure how that happened, will make sure it is included in the revisions*

Revisions reviewer 2

1. The authors have tried to address my earlier concern by adding the "combiSV" sub-section under the Method section. Unfortunately, this sub-section still doesn't provide enough detail. They should extend this sub-section further and provide more detail, particularly related to the equation mentioned in the added text.

*I should not have mentioned the word “equation”, because that is not describing the method accurately. Instead, when an SV is called by multiple callers, it will prioritize one SV caller for each of the stats (genotype, position, length and type). Some additional SVs that were detected by one caller will also be included when the false positive rate was significantly low (for example for homozygous SVs from SVIM). I extended the paragraph to explain this more clearly. Every detail can also be found in the code, which is publicly available.*

2. Can the authors clarify whether the precision and recalls score of SURVIVOR reported in Table2 is the highest score for any combination of 3 SV tools used in the SURVIVOR method?

*That should have been tested indeed. We tried 9 combinations for SURVIVOR and combiSV. The combination (pbsv, cuteSV and Sniffles) we used in Table 2 was the best performing combination for both combiSV and SURVIVOR on the real GIAB dataset. But it was also the worst combination (because of a low recall) for SURVIVOR on the simulated dataset of Nanopore. This is because the GIAB dataset contains only insertions and deletions in relatively “easy” regions. Most of these SVs will be detected by all tools, so best to combine the 3 tools with the least false positives. The simulated dataset contains also SVs in complex regions, which are better detected by less precise callers. Therefore, SURVIVOR need at least one less precise caller with a high recall. We removed the column of the 3 callers in table 2 and added a table to the main article and one to the supplementary materials were 9 combinations of SURVIVOR and combiSV are compared to each other on the simulated and the GIAB dataset. From all 18 combinations, combiSV had the better F-score for 17 combinations. The combination wherefore SURVIVOR scored better is SVIM, NanoSV and NanoVar, which does not belong to any of the better performing combinations and will therefore be of no relevance. We also designed combiSV that it should have at least pbsv, cuteSV or Sniffles in the combination, we did not optimize the algorithm for that one combination without all 3. Furthermore, the total score that includes accuracy of the calls was always better for combiSV. SURVIVOR’s performance depends strongly on which callers are combined and even the order in how you give the 3 callers has a significant impact on the results. On the other hand, combiSV has a very similar performance for all combinations and the order has no impact.*

**Third round of review**

**Reviewer 1**

I want to thank the authors for addressing all my comments in the previous round, and I think clarity of this manuscript has been significantly improved. I just have one minor comment, with which fixed the manuscript should be good to go.

Minor Comment: The authors put a really nice discussion of the pros and cons of "total score" in their response to reviewers' comments, it would be best if the downside of total score is also discussed in the discussion section, so readers could have a comprehensive view of the performance comparison results.

**Reviewer 2**

Authors have addressed all my concerns in the revised manuscript.
